# Lef1 ablation alleviates cartilage mineralization following posttraumatic osteoarthritis induction

**DOI:** 10.1073/pnas.2116855119

**Published:** 2022-05-20

**Authors:** Jinan Elayyan, Idan Carmon, Lital Zecharyahu, George Batshon, Yonathan H. Maatuf, Eli Reich, Maitena Dumont, Leonid Kandel, Michael Klutstein, Mona Dvir-Ginzberg

**Affiliations:** ^a^Institute of Biomedical and Oral Research, Faculty of Dental Medicine, Hebrew University of Jerusalem, Jerusalem, 9112102 Israel;; ^b^Koret School of Veterinary Medicine, Robert H. Smith Faculty of Agricultural, Food, and Environment, Hebrew University of Jerusalem, Rehovot, 76100 Israel;; ^c^Orthopedic Unit, Hebrew University–Hadassah Medical Center, Jerusalem, 9112102 Israel;; ^d^Chromatin and Aging Research Lab, Faculty of Dental Medicine, Hebrew University of Jerusalem, Jerusalem, 9112102 Israel

**Keywords:** cartilage, mineralization, osteoarthritis, meniscus, Lef1

## Abstract

Cartilage mineralization is a tightly controlled process, imperative for skeletal growth and fracture repair. However, in osteoarthritis (OA), cartilage mineralization may impact the joint range of motion, inflict pain, and increase chances for joint effusion. Here we attempt to understand the link between inflammation and cartilage mineralization by targeting Sirtuin 1 (SIRT1) and lymphoid enhancer binding factor 1 (LEF1), both reported to have contrasting effects on cartilage. We find that inflammatory-dependent cleavage of SIRT1 or its cartilage-specific genetic ablation, directly enhanced LEF1 expression accompanied by a catabolic response. Applying a posttraumatic OA (PTOA) model to cartilage-specific *Sirt1* nulls displayed severe OA, which was accompanied by synovitis, meniscal mineralization, and osteophyte formation of the lateral joint compartment. Alternatively, cartilage-specific *Lef1* nulls presented reduced lateral mineralization, OA severity, and local pain. Differential gene expression analysis revealed that Lef1 ablation reduced nuclear factor kappa-light-chain-enhancer of activated B cells (*NF-κB*) and Toll-like receptor (*Tlr*) pathways, while enhancing SRY-Box transcription factor 9 (*Sox9*) and cartilaginous extracellular matrix genes. The results support a link between inflammation and *Lef1*-dependent cartilage mineralization, mediated by the inactivation of *Sirt1*. By ablating *Lef1* in a PTOA model, the structural and pain-related phenotypes of OA were reduced, in part, by preventing cartilage mineralization of the lateral joint compartment, partially manifested by meniscal tissue mineralization. Overall, these data provide a molecular axis to link between inflammation and cartilage in a PTOA model.

## Significance

Cartilage mineralization is imperative in various processes such as skeletal growth and fracture repair. However, this process may also be pathological, as in the case of the degenerative joint disease, osteoarthritis (OA). Using a posttraumatic OA model (PTOA), we find that cartilage-specific *Sirt1* genetic nulls caused severe synovitis and mineralization of the lateral joint compartment, due to augmented *Lef1* gene expression. Conversely, cartilage-specific *Lef1* nulls exhibited impaired synovitis and mineralization of the lateral joint, accompanied by a reduction of local pain. Consistently, transcriptomic profiles of *Lef1*-ablated chondrocytes exhibited enhanced anabolism, yet impaired pathways related to calcification and inflammation. Accordingly, cartilage mineralization of the lateral joint compartment relies on amplified inflammatory pathways, contributing to articular damage following PTOA.

The capacity of cartilage tissue to mineralize is an integral part of the physiology and pathology in mammals. In fact, the process of skeletal growth relies upon endochondral mineralization, wherein chondrocytes (resident cartilage cells) rapidly proliferate and undergo hypertrophy and mineralization, enabling longitudinal epiphysial growth and remolding of lamellar bone (1). In addition to their developmental role, cartilage hypertrophy and mineralization are required for long bone fracture repair, yet are also associated with joint diseases, such as osteoarthritis (OA) (2–5). Interestingly, cartilage hypertrophy and mineralization are often accompanied by a catabolic response, often preceded by proinflammatory induction (2–5). For example, during fracture repair in adults, the requirement for callus mineralization has been reported to be driven by inflammation, within the first 24 h of trauma (2). Similarly, during OA pathology, synovitis accompanies articular damage and ectopic cartilage mineralization, appearing in the form of osteophytes and/or mineralization of meniscal tissue (5). These structural changes to the joint, inflict loss of joint mobility, contribute to joint effusion, and are accompanied by severe pain in patients afflicted with OA (3, 4). While much is known regarding mechanisms driving articular cartilage damage, breakdown, and catabolism during inflammation, little is known about the mechanisms driving cartilage mineralization, within the context of joint inflammation.

Specifically, rheumatic diseases, including OA, are often accompanied by increased tumor necrosis factor alpha (TNF-α) and interleukin 1beta (IL-1β), both shown to contribute to cartilaginous matrix breakdown, by up-regulating cartilage catabolic enzymes (e.g., matrix metalloproteinases [*MMP*s] and disintegrin and metalloproteinase with thrombospondin motifs [*ADAMTS*]), often leading to progressive articular cartilage damage and ectopic mineralization of the joint surfaces (3–6). Similarly, low-dose TNF-α administration was reported to contribute to fracture repair and callus mineralization in a murine model (7). Moreover, a subset of individuals with juvenile rheumatoid arthritis were reported to exhibit impaired growth, indicating that autoimmune disease could affect the dynamic processes required for epiphyseal growth (8). This albeit circumstantial evidence highlights the possibility that inflammation not only drives cartilage catabolism and breakdown, but also orchestrates its hypertrophy and mineralization. Therefore, our aim herein was to examine a possible molecular link between inflammation and cartilage hypertrophy and mineralization using OA as a disease model.

To further inspect this notion, we evaluated WNT/β-catenin signaling pathway–inducing bone mineralization, since within the context of OA, its attributes on cartilage mineralization were less conclusive (9), particularly some WNT agonists, such as WNT3a (10) and WNT5a (11) were associated with OA pathophysiology, while others such as WNT16 and WNT11 were proven to prevent OA (12, 13). Moreover, several reports support the finding that activation of β-catenin aggravates OA (14), while conversely other reports demonstrate similar OA induction, by inhibiting β-catenin in articular chondrocytes (15). These data question the role of the canonical WNT pathway in driving OA structural damage and potentially imply that alternative, noncanonical WNT signaling may evoke OA pathogenesis, possibly by inflammatory insults. In line with this notion, Yun et al. reported that IL-1β stimulation of chondrocytes augmented LEF1 (i.e., a transcription factor of the WNT/β-catenin pathway), inducing the nuclear translocation of nuclear factor kappa-light-chain-enhancer of activated B cells (NF-κB) in stimulated chondrocytes (16). Moreover, LEF1 was reported to activate MMP13 by binding a 3′ regulatory site on the gene locus (17). These reports cumulatively support the notion that inflammation may trigger cartilage breakdown, via noncanonical WNT signaling, a hypothesis we further examine herein in relation to *Sirt1* cleavage and inactivation. This mechanistic assumption, supported by previous data, has shown that SIRT1 blocked LEF1 and subsequent MMP13 expression, while cleavage of SIRT1 correlated with increased LEF1 and MMP13 expression (18).

Beyond cartilage metabolism, *Lef1* has been reported to be required for proper development and bone turnover, in systemic knockouts or haploinsufficient mice models (refs. 19 and 20, respectively), as well as for promoting tooth development via odontoblastic differentiation (21). Moreover, the importance of *Lef1* and *β-catenin* signaling has been highlighted in atherosclerosis development (22) and vascular smooth muscle mineralization (23), potentially providing a broader impact to this signaling circuit. Cumulatively, these observations support the notion that *Lef1*, a member of the WNT signaling pathways, plays central roles in regulating tissue mineralization, a mechanistic assumption we aim to elucidate in this report.

## Materials and Methods

### Human Cell Cultures.

Human chondrocytes were isolated from end-stage OA patients who underwent total knee arthroplasty, (*n* = 25, mean age 71 y, mean body mass index 30.5 kg/m2). Written informed consent was obtained from all patients prior to the procedure. The study protocol included clinically established end-stage OA, based on a Kellgren and Lawrence (KL) score of 3 to 4, according to radiographic evidence of the affected knee joint. The full study protocol was approved by the institutional ethics committee of Hadassah Medical Center (institutional approval 04880-09-HMO). Collected joints were dissected for intact cartilage and articular chondrocytes were isolated and cultured, as previously described (24). Detailed protocols related to human cultures are provided in *SI Appendix*, SI_1.

### Mice Experimental Procedures.

Mice-related experimental procedures were carried out in accordance with NIH committees for animal use and care (Animal Research Advisory Committee guidelines) and based on Association for Assessment and Accreditation of Laboratory Animal Care International guidelines. The Hebrew University Institutional Animal Care and Use Committee approved the study’s protocols (MD-12-13383-4, MD-14-14172-2, and MD-18-15660-1). Mice were subjected to 12-h light/dark cycles and received food and water ad libitum.

Transgenic strains (C57BL/6 background) included a cartilage-specific Sirt1-knockout (KO) mouse (*ATC Sirt1^fl/fl^*), which was generated by breeding *Sirt1^fl/fl^* (The Jackson Laboratory, 008041) with *ATC* transgenic mice (provided by Veronique Lefebvre, Children's Hospital of Philadelphia Research Institute, Philadelphia, PA), as previously described (25, 26). Similarly, *ATC* transgenic mice were crossed with *Lef1^fl/fl^* (provided by Hai-Hui Xue, University of Iowa, Iowa City, IA) (27) to generate cartilage-specific Lef1-knockout mice (*ATC Lef1^fl/fl^*). Notably, *ATC* transgenic mice express an Agc1 enhancer-driven, tetracycline-inducible Cre (ATC) transgene (25), which we induced in 3-mo-old adult mice, 2 wk prior to posttraumatic OA (PTOA) procedures, or in pregnant dams between E11.5 to E17.5 gestational days, by adding doxycycline (0.8 mg/400 mL; Biobasic, catalog [Cat.] No. 24390-14-5) to the drinking water. Additional PTOA experiments were carried out with C57BL/6 (The Jackson Laboratory) female, wild-type (WT) mice at 3 mo old. Briefly, following 4 wk PTOA, mice were injected intraarticularly (IA) for the following 4 wk, twice a week, as follows: Vehicle (20% v/v dimethyl sulfoxide (DMSO) in phosphate-buffered saline), Sirt1 activator (81 μM SRT1720; ApexBio, Cat. No. A8239), or Cathepsin B inhibitor (600 μM CA074 methyl ester (CA074me); BioVision, Cat. No. 2772), or a combination of both compounds (CA074me 300 μM and SRT1720 40.5 μM). After 8 wk PTOA, the mice were euthanized by cervical dislocation following anesthesia (ketamine and xylazine 200 mg/kg).

Detailed protocols related to mice strains and analysis are provided in *SI Appendix*, SI_1. *SI Appendix*, SI_1 additionally includes all materials and methods applied in this report, as well as explanations related to *SI Appendix*, SI_2–SI_8 and SI_10 and Dataset SI_9.

## Results

### Sirt1 Directly Represses Lef1 Expression in Chondrocytes.

**Fig. 1.**
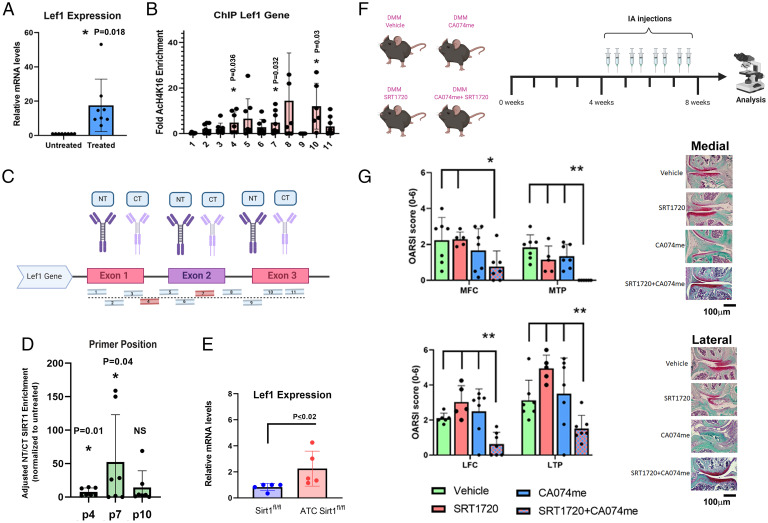
Sirt1 directly represses Lef1 gene expression. Human chondrocytes were grown to passage 1 and treated with IL-1β (5 ng/mL) and TNF-α (50 ng/mL) for 24 h and analyzed for (*A*) mRNA expression of full-length LEF1 (*n* = 8). (*B*) Treated human chondrocytes normalized to untreated chondrocytes were analyzed for acetylation of H4K16 (AcH4K16) on 11 intergenic regions of the human LEF1 gene promoter (*n* = 8). (*C*) Scheme of primers (denoted as numbers) flanking on the LEF1 intergenic locus spanning between promoters 1 and 2. (*D*) AcH4K16-enriched sites detected in *B* (i.e., p4, p7; “‎p” denoting primer sites) and nonsignificance (NS) at p10, were analyzed via ChIP for NT and CT SIRT1 upon the LEF1 gene (*n* = 8). (*E*) Costal cartilage of *Sirt1^fl/fl^* and *ATC Sirt1^fl/fl^* mice were plated and analyzed for *Lef1* gene expression (*n* = 5). (*F* and *G*) Combination treatment of PTOA (*n* ≥ 5, females, 3 mo old). (*F*) Experimental illustration of IA treatment beginning after 4 wk of PTOA and administred twice a week with vehicle, SRT1720 and\or CA074me, starting at 4 wk PTOA until 8 wk. (*G*) Osteoarthritis Research Society International (OARSI) scoring (0 to 6) for medial tibial plateau (MTP), lateral tibial plateau (LTP), medial femoral condyle (MFC), or lateral femoral condyle (LFC).

Previous work from our group has shown that SIRT1 cleavage and loss of activity are related to OA by inducing cartilage catabolic expression (18). Specifically, we found that loss of SIRT1 is associated with increased LEF1 expression and protein levels, which was later directly reported to activate MMP13 gene expression (17). Here we sought to decipher whether SIRT1 directly represses LEF1 gene expression by binding its promoter sites. Initially, our data show that following cytokine stimulation of human OA–derived chondrocytes, there is a significant increase in LEF1 expression (Fig. 1*A*). To decipher whether this enhanced LEF1 expression is dependent on SIRT1, we carried out a chromatin immunoprecipitation (ChIP) assay to screen for enhanced acetylation of H4K16 (AcH4K16, a SIRT1 histone substrate) in the multiple promoter loci that have been reported to regulate the expression of the full-length transcript of LEF1 (28). ChIP analysis for 11 regulatory loci of LEF1 detected enhanced acetylation of H4K16 in primer sites p4, p7, and p10 following cytokine stimulation, indicating these regions are euchromatic and likely contribute to LEF1 gene activation (Fig. 1 *B* and *C*). Should SIRT1 inhibit LEF1 expression, these regions may occupy inactive SIRT1, which is devoid of deacetylase activity (26). To assess whether the cleaved and inactivated SIRT1 variants occupy these sites, we tested p4, p7, and p10 for N-terminal (NT) or C-terminal (CT) domains of SIRT1 via ChIP analysis (Fig. 1*D*). The data display higher enrichment for NT vs. CT SIRT1 in p4 and p7 sites following cytokine stimulation. Therefore, SIRT1 may be cleaved and inactivated within the cell nucleus, affecting its regulatory capacity in genomic sites as LEF1. Finally, costal or articular chondrocytes were isolated from cartilage-specific *Sirt1* null mice (i.e., *ATC Sirt1 ^fl/fl^*) vs. *Sirt1^fl/fl^* and tested for *Lef1* expression, confirming that lack of *Sirt1* enhanced *Lef1* expression (Fig. 1*E* and *SI Appendix*, SI_3*G*), which establishes a direct and inverse link between SIRT1 activity and LEF1 expression.

To further understand the physiological relevance of Sirt1 cleavage and inactivation in directing OA, we employed a PTOA model, which was administered with vehicle control, SRT1720 (a Sirt1 activator), CA074me (a cathepsin B inhibitor), and both SRT1720 and CA074me in combination at 4 wk PTOA and until euthanizing the animals at 8 wk PTOA (e.g., intraarticular administration, twice a week, as in Fig. 1*F*). The data show that the combination treatment reduced OA severity significantly vs. each individual treatment or vehicle control (Fig. 1*G*). Furthermore, combination treatment reduced osteophyte formation in the lateral femoral condyle (*SI Appendix*, SI_11*A*), accompanied by reduced pain (*SI Appendix*, SI_11*B*) and synovitis mainly in the medial compartment (*SI Appendix*, SI_11 *C* and *D*). Histological analysis revealed that the combination treatment exhibited reduced LEF1 levels vs. vehicle control (*SI Appendix*, SI_11*E*), thus possibly supporting the notion that the combination treatment effectively halted OA by repressing LEF1 levels. Cumulatively, the data support the idea that both *Sirt1* cleavage and inactivation result in *LEF1* expression. Pharmacological modulation of *Sirt1* cleavage may prevent OA, in part by repressing *Lef1* expression.

### PTOA ATC Sirt1^fl/fl^ Mice Display Enhanced Mmp13 Levels and Synovitis of the Lateral Joint Compartment.

Previous data established that PTOA *ATC Sirt1^fl/fl^* mice exhibit augmented articular cartilage damage, especially in the lateral compartment compared to *Sirt1^fl/fl^* (26). To further characterize the joint damage, we assessed PTOA synovitis for *ATC Sirt1^fl/fl^* and *Sirt1^fl/fl^* (*SI Appendix*, SI_12). Indeed, synovial thickness was two-fold higher in the lateral tibial plateau (LTP) *of ATC Sirt1^fl/fl^* vs. *Sirt1^fl/fl^* PTOA mice (*SI Appendix*, SI_12*A*). Staining for F4/80 synovial macrophages confirmed their relative abundance in the lateral compartment of *ATC Sirt1^fl/fl^* vs. *Sirt1^fl/fl^* PTOA mice (*SI Appendix*, SI_12*B*). Immunohistochemical analysis of the lateral menisci revealed that *ATC Sirt1^fl/fl^* exhibit stronger MMP13 staining vs. *Sirt1^fl/fl^* PTOA mice (*SI Appendix*, SI_12*C*), which was in line with increased *Mmp13* expression in costal and articular chondrocytes from *Sirt1* ablated mice (*SI Appendix*, SI_12*D* and SI_3*G*).

### PTOA ATC Sirt1^fl/fl^ Mice Display Enhanced Osseous Remodeling (LOR) of the Lateral Joint Compartment.

**Fig. 2.**
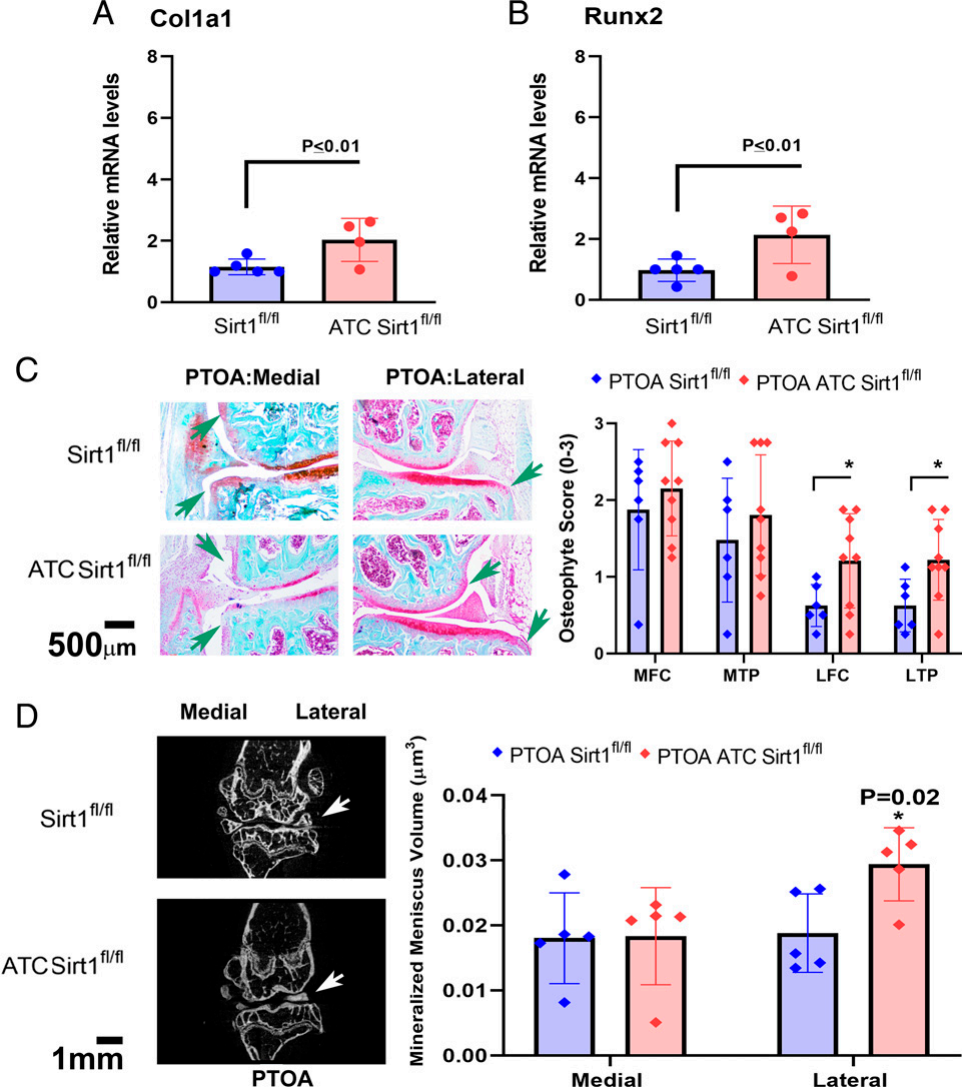
PTOA-induced *ATC Sirt1^fl/fl^* mice display increased mineralization of the lateral compartment. Costal chondrocytes were isolated from E17 *Sirt1^fl/fl^* and *ATC Sirt1^fl/fl^* mice and analyzed for (*A*) *Col1a1* and (*B*) *Runx2* gene expression (*n* = 5). (*C*) Representative images of joint sections stained with Safranin-O/Fast green from PTOA *Sirt1^fl/fl^* and *ATC Sirt1^fl/fl^* mice (*Sirt1^fl/fl^ n* = 6; female *n* = 3 and male *n* = 3; *ATC Sirt1^fl/fl^ n* = 9; female *n* = 4 and male *n* = 5), exhibiting osteophyte formation (green arrows). The plot to the *Right* depicts osteophyte scores (0 to 3) for MTP, LTP, MFC, or LFC. (*D*) Longitudinal two-dimensional (2D) sections were extracted from three-dimensional (3D) reconstructions of PTOA joints following microcomputed tomography (µCT) scanning. Results in images (*Left*) exhibit calcified menisci (white arrows) of *Sirt1^fl/fl^* and *ATC Sirt1^fl/fl^* mice (*n* = 6; female *n* = 3 and male *n* = 3). Mineralized menisci volume (μm^3^) is plotted on the *Right*.

Given that WNT signaling is often attributed to chondrocyte hypertrophy (5, 29), we examined *Col1a1* and *Runx2* expression, which displayed enhanced expression in costal chondrocytes from *ATC Sirt1^fl/fl^* vs. *Sirt1^fl/fl^* (Fig. 2 *A* and *B*, respectively). Articular chondrocytes exhibited unchanged *Col1a1* expression, yet increased but insignificant *Runx2* expression levels upon Sirt1 ablation (*SI Appendix*, SI_3*G*), potentially due to the opposing effects of *Sirt1* on *β-catenin* signaling [i.e., repressing *Lef1* (18), while stabilizing *β-catenin* (30)]. To examine this on a physiological level, we next monitored osteophyte numbers for all compartments of both genotypes in PTOA mice. Our results show significantly higher osteophyte numbers in the lateral compartment of *ATC Sirt1^fl/fl^* vs. *Sirt1^fl/fl^*, PTOA mice (Fig. 2*C*), which were unaffected in sham controls (*SI Appendix*, SI_13*A*). Next, we examined PTOA meniscal mineralization and observed increased lateral meniscal mineralization in the *ATC Sirt1^fl/fl^* compared to *Sirt1^fl/fl^* mice (Fig. 2*D*), with unaffected trends in sham controls (*SI Appendix*, SI_13*B*). Similarly, mice treated with combination treatment (SRT1720 and CA074me; experimental setup in Fig. 1*F*) exhibited reduced collagen I staining in the external region of the lateral meniscus vs. vehicle control of PTOA mice (*SI Appendix*, SI_11*F*), indicating that *Sirt1* cleavage and inactivation, similar to its genetic ablation, promotes cartilage mineralization of the lateral joint compartment. Overall, the data suggest that the lateral compartment not only undergoes articular damage in the PTOA *ATC Sirt1^fl/fl^* mice, but it also displays enhanced synovitis and lateral osseous remodeling (LOR) (i.e., osteophyte formation and mineralization of the meniscus).

### Lef1 Ablated Chondrocytes Exhibit Reduced Mmp13, Col1a1, and Runx2.

Previous reports indicated that systemic Lef1 KO was lethal (2 wk after birth) and displayed reduced size and significant developmental defects (19). Moreover, *Lef1* haploinsufficient mice were reported to display low bone mass and reduced bone turnover (20). To assess the physiological effects of Lef1 in cartilage biology, we generated a unique *ATC* *Lef1^fl/fl^* mouse strain. As a first step, we characterized the skeletal features of E17 embryos from Lef1 transgenes of pregnant dams. Surprisingly, our data did not display significant differences in the gross size, hindlimb, femur, and skull of the *ATC Lef1^fl/fl^* vs. *Lef1^fl/fl^* littermates (*SI Appendix*, SI_14 *A*–*C*), nor were the mice lethal perinatally.

**Fig. 3.**
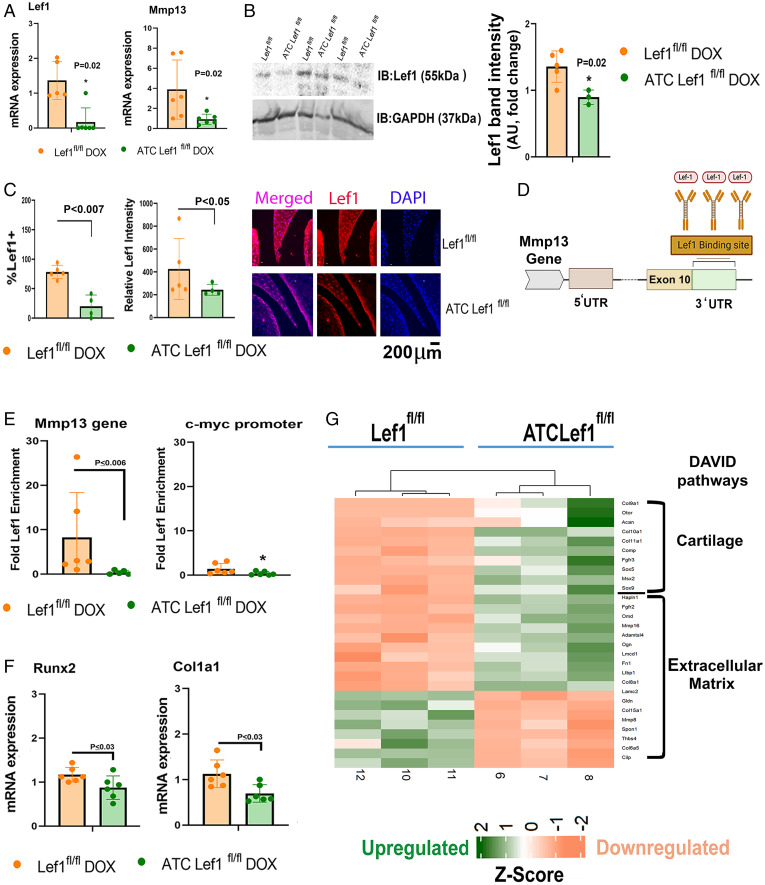
Genetic Lef1 ablation from chondrocytes reduced *Mmp13* expression. Costal chondrocytes were isolated from E17 *Lef1^fl/fl^* and *ATC Lef1^fl/fl^* mice and analyzed for the expression of (*A*) *Lef1* and *Mmp13* (*n* = 6), as well as (*B*) Immunoblotted for Lef1 (*n* = 4 each) and quantified for band intensity (*Right*). (*C*) Immunostained sections for LEF1 (red) in 8-wk post sham-treated mice (i.e., *ATC Lef1^fl/fl^* or *Lef1^fl/fl^*). Graphs indicate percent of cells positive for LEF1 or intensity of LEF1 staining, in a given field. *Right* displays representative images of the lateral menisci *Lef1^fl/fl^* (*n* = 5) and *ATC Lef1^fl/fl^* (*n* = 4). (*D*) Scheme of 3′ UTR *Lef1*-binding site on the *Mmp13* gene locus. (*E*) ChIP analysis for *Lef1* enrichment on the *Mmp13* gene (*Left*; *n* = 6). *Right* graph exhibits a positive control for the *c-myc* gene bearing the putative Lef1 DNA-binding site. (*F*) Costal chondrocytes were isolated, as indicated in *A*, and assessed for the expression of *Runx2* and *Col1a1* (*n* = 6). (*G*) Differentially expressed genes are presented as the Z-score value based on LFC, classified into cartilage and extracellular matrix (ECM) genes (*SI Appendix*, SI_6), according to DAVID annotation (*n* = 3). Green panels are up-regulated genes; orange panels are down-regulated genes.

We next obtained costal and articular chondrocytes from *ATC Lef1^fl/fl^* and *Lef1^fl/fl^* littermates and confirmed that upon culture with doxycycline, *Lef1* and its target *Mmp13* gene were both significantly reduced in *ATC Lef1^fl/fl^* vs. *Lef1^fl/fl^* chondrocytes (Fig. 3*A* and *SI Appendix*, SI_3*H*, respectively). Reduced LEF1 protein levels were further confirmed via immunoblot analysis for costal chondrocytes (Fig. 3*B*) and immunofluorescence for meniscal cartilage tissue (Fig. 3*C*; 8 wk following sham procedure, 5 mo old). To test the impact of *Lef1* ablation on the *Mmp13* gene, we performed a ChIP analysis for Lef1 on the 3′ untranslated region (UTR) of murine *Mmp13* gene (Fig. 3*D*), which displayed reduced enrichment in the *ATC Lef1^fl/fl^* vs. *Lef1^fl/fl^* chondrocytes (Fig. 3*E*). Similarly, ChIP for *Lef1* on the c-myc promoter, containing putative *Lef1* DNA-binding sites, expectedly exhibited reduced enrichment of *Lef1* protein levels in *ATC Lef1^fl/fl^* (Fig. 3*E*, *Right* graph), confirming the genetic ablation. Finally, ablation of *Lef1* in costal chondrocytes resulted in reduced expression of *Col1a1* and *Runx2* (Fig. 3*F*), a result which was in line with expression trends of articular chondrocytes, albeit a statistically insignificant reduction for *Col1a1* (*SI Appendix*, SI_3H).

RNA sequencing (RNA-Seq) from costal chondrocytes derived from *ATC Lef1^fl/fl^* vs. *Lef1^fl/fl^* unveil additional gene targets that are affected by *Lef1* ablation in costal chondrocytes (*SI Appendix*, SI_6: Tables A–D and Fig. 3*G*). In particular, *Lef1* ablation resulted in up-regulated “*Chondrogenic*” and “*Cartilage*” gene clusters (*SI Appendix*, SI_6: Table A). The chondrogenic transcription factors *Sox9* and *Sox5* were significantly elevated upon Lef1 ablation (*SI Appendix*, SI_6: Table A), which was accompanied by enhanced cartilage-specific ECM encoding genes (i.e., *Acan*, *Col9a1*, and *Col11a1*). STRING network analysis of gene ontology (GO) enrichment clusters exhibited increased carbohydrate processing networks (*SI Appendix*, SI_5*C* and SI_8: Table C), including *Carbohydrate Sulfotransferase 1* (*Chst1*) and *Hyaluronan and Proteoglycan Link Protein 1 (Hapln1*), required for proteoglycan assembly. These data coincide with reports showing that WNT signaling (i.e., *Lef1/Tcf/β-catenin*) abrogates the chondrogenic activity of *Sox9* and its chondrogenic targets (31, 32).

Interestingly, the number of “*Catabolic*” genes up-regulated due to *Lef1* ablation is lower (*SI Appendix*, SI_6: Table C) than the number of genes increased under the “*Fibrous Collagens*” classification (*SI Appendix*, SI_6: Table B), implying that *Lef1* ablation elicited more matrix production than breakdown in cartilage (*SI Appendix*, SI_6: Table A and D and heatmap in Fig. 3*G*). Contrary to our evidence of reduced *Col1a1* and *Runx2* detected via PCR in *Lef1*-ablated chondrocytes (Fig. 3*F*), *Col10a1* was significantly up-regulated among the fibrillar collagens (*SI Appendix*, SI_6: Table B), indicating chondrocyte hypertrophy may occur during *Lef1* ablation. Intriguingly, this hypertrophic capacity was reported to be driven by *Sox9* (25), which is significantly enhanced upon *Lef1* ablation (*SI Appendix*, SI_6: Table A), implying that *Sox9* transcriptional versatility may depend on its interaction with various transcriptional regulators. Moreover, it is possible that subsequent to hypertrophy, *Lef1* may act independently in inducing cartilage mineralization, by directly regulating *Runx2* expression (33).

### PTOA ATC Lef1^fl/fl^ Mice Exhibit Reduced OA-Associated Structural Joint Damage.

**Fig. 4.**
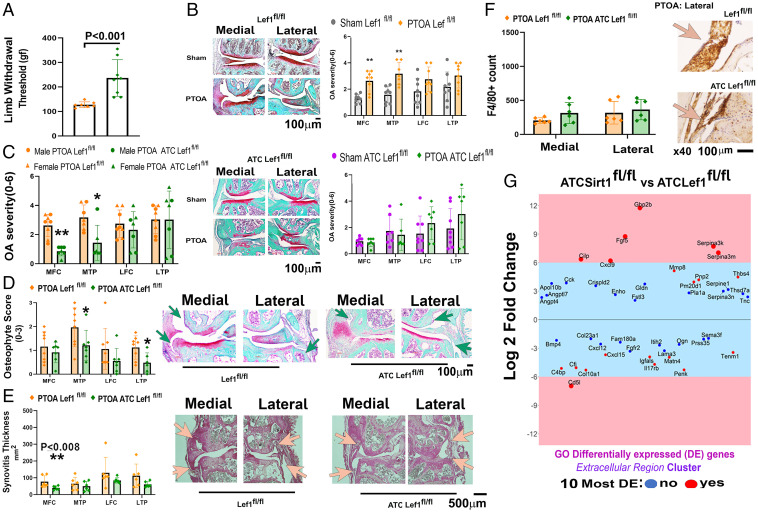
Cartilage-specific Lef1-ablated mice exhibit less severe posttraumatic OA (PTOA) phenotype. Three-month-old *Lef1^fl/fl^* and *ATC Lef1^fl/fl^* mice were treated for 2 wk with doxycycline and subjected to PTOA or sham procedures. (*A*) Knee hyperalgesia was measured by using a PAM device for PTOA *Lef1^fl/fl^* and *ATC Lef1^fl/fl^* mice, 8 wk PTOA (*n* = 8; female *n* = 4 and male *n* = 4). (*B*) Safranin O/Fast green staining of joints from *Lef1^fl/fl^* (*Upper* depictions and graph) and *ATC Lef1^fl/fl^* mice (*Lower* depictions and graph). (*C*) OA severity ranking between PTOA *Lef1^fl/fl^* (*n* = 8; female *n* = 4 and male *n* = 4) and *ATC Lef1^fl/fl^* (*n* = 7; female *n* = 4 and male *n* = 3). PTOA *Lef1^fl/fl^* and *ATC Lef1^fl/fl^* (*n* = 8) mice were analyzed for (*D*) osteophyte formation (same sample size as *C*); green arrows pointing at osteophytes. (*E*) Synovial thickness (pink arrows pointing at synovial lining); and (*F*) F4/80 positive macrophages in synovial lining with lateral representative images. (*G*) Transcriptomic analysis for costal chondrocytes from *ATC Sirt1^fl/fl^ (n =* 2) vs. *ATC Lef1 ^fl/fl^* (*n* = 3) were analyzed and found to exhibit 178 up-regulated and 96 down-regulated genes. GO enrichment gene cluster was obtained for the “‎Extracellular Region” classification, to determine differentially expressed genes that may ‎be secreted from cartilage (*SI Appendix*, SI_10). This cluster exhibited 23 up-regulated and 20 down-regulated genes (*SI Appendix*, SI_10). The 10 most differentially expressed genes are marked in red dots. Specific up-regulated genes (over plus sixfold) and down-regulated genes (less than minus sixfold) are exhibited on a pink background within the plot.

Three-month-old *ATC Lef1^fl/fl^* and *Lef1^fl/fl^* mice were subjected to PTOA and monitored for joint pain using a pressure application measurement (PAM) device after 8 wk PTOA. PTOA *ATC Lef1^fl/fl^* mice exhibited increased pain thresholds indicating that local pain sensitivity of the joint is lower than for *Lef1^fl/fl^* mice (Fig. 4*A*). While PTOA *Lef1^fl/fl^* exhibited increased articular cartilage damage in the medial compartment (Fig. 4 *B*, *Upper*) vs. shams, *ATC Lef1^fl/fl^* mice exhibited similar articular cartilage damage for all compartments vs. shams (Fig. 4 *B*, *Lower*). Comparing the structural features of PTOA between the genotypes revealed cartilage damage is significantly reduced in the medial compartment of *ATC Lef1^fl/fl^* vs. *Lef1^fl/fl^*, with unchanged damage to articular cartilage of the lateral compartment (Fig. 4*C*), a trend also confirmed upon addition of two sequentially spaced graded sections (*SI Appendix*, SI_15*A*). Impaired serum NT/CT Sirt1 ratio provided additional support of reduced PTOA joint damage phenotype of the *ATC Lef1^fl/fl^* vs. *Lef1^fl/fl^* (*SI Appendix*, SI_15*B*, *Left* graph) (26), which was also associated with down-regulated *cathepsin B* in the *ATC Lef1^fl/fl^* (*SI Appendix*, SI_8; Table D and SI_5*D*). Similarly, serum NT/CT Sirt1 reduction was observed in the combination treatment vs. vehicle control (*SI Appendix*, SI_15*B*, *Right* graph).

Monitoring osteophyte formation revealed that *ATC Lef1^fl/fl^* mice exhibit reduced osteophyte scores in tibial (medial and lateral) compartments, compared to PTOA *Lef1^fl/fl^* mice (Fig. 4*D*). We next assessed subchondral tibial bone thickness and bone volume over total volume (%BV/TV), which did not display significant differences among the genotypes for sham (*SI Appendix*, SI_15*C*), yet displayed enhanced lateral subchondral %BV/TV and plate thickness for PTOA *ATC Lef1^fl/fl^* vs. *Lef1^fl/fl^* (*SI Appendix*, SI_15 *D* and *E*). This change in the subchondral bone may insinuate that Lef1-mediated cartilage levels may regulate bone remodeling following PTOA, which is in line with enhanced *Tnfrsf11b* (osteoprotegrin) levels detected in *Lef1*-ablated chondrocytes (Dataset SI_9).

Interestingly, PTOA mice did not display variations in synovitis among the *ATC Lef1^fl/fl^* and *Lef1^fl/fl^* (Fig. 4 *E* and *F*) for most joint compartments. To understand this discrepancy with the *Sirt1* transgenes, which exhibited enhanced lateral synovitis upon *Sirt1* ablation (*SI Appendix*, SI_12 *A* and *B*), we compared the transcriptome of *ATC Sirt1^fl/fl^* vs. *ATC Lef1^fl/fl^* (*SI Appendix*, SI_3*E*, principal component analysis plot; *SI Appendix*, SI_3*F*, volcano plot) and focused on the “extracellular region” GO gene cluster containing differentially expressed (DE) genes that may be secreted from chondrocytes (*SI Appendix*, SI_10). Fig. 4*G* exhibits six up-regulated genes (i.e., *Gbp2b*, *Fgf5*, *Serpina-3k/-3m*, *Clip*, and *Cxcl9*) with a sixfold increase for the *ATC Sirt1^fl/fl^* vs. *ATC Lef1^fl/fl^* chondrocytes and one equivalent down-regulated gene (i.e., *Cd5l*). Of the up-regulated genes, *Gbp2b*, *Cxcl9*, and *Cilp* were implicated in immune activation (34–37), indicating that lack of Sirt1 in cartilage could prompt synovitis via chondrocyte secretome. Notably, the combination treatment exhibited significantly reduced synovial characteristics in the medial compartment (*SI Appendix*, SI_11 *C* and *D*); in contrast, the ATC Sirt1^fl/fl^ transgenes exhibited lateral synovitis (*SI Appendix*, SI_12 *A* and *B*), potentially indicating that the development of lateral synovitis relies on Sirt1 expression, as well as its protein activity.

**Fig. 5.**
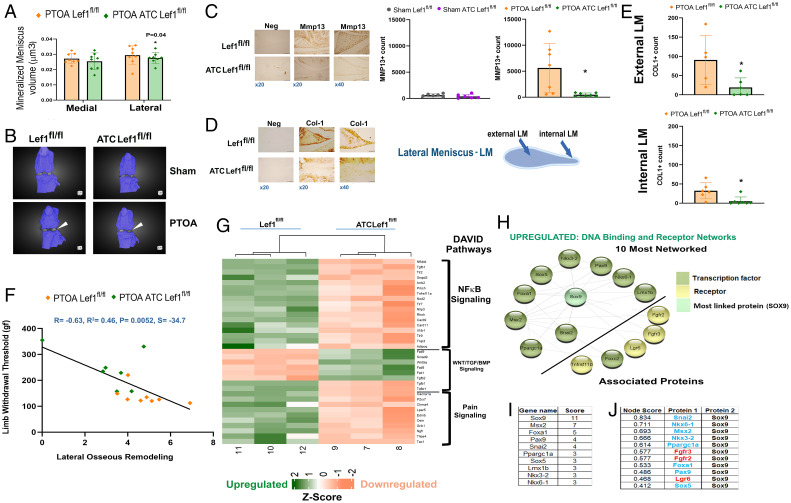
PTOA Lef1-ablated mice exhibit reduced osseous-like phenotypes in the lateral compartment. The 3D µCT joint reconstructions of PTOA *Lef1^fl/fl^* and *ATC Lef1^fl/fl^* mice were analyzed for mineral content (μm^3^) of the medial and lateral menisci (*A*). Representative 3D images show calcified menisci colored white (white arrows) of PTOA and sham *Lef1^fl/fl^* and *ATC Lef1^fl/fl^* mice (*B*). PTOA *Lef1^fl/fl^* and *ATC Lef1^fl/fl^* mice (*n* = 8; female *n* = 4 and male *n* = 4) were analyzed for (*C*) MMP13 staining of the lateral menisci and ImageJ quantification (*Right* graphs sham and PTOA). *Left* image depiction of immunostaining shows a negative control (denoted “Neg”). (*D*) Collagen type I immunostaining and (*E*) quantification for internal and external fields of the lateral menisci (“LM”; illustrated to the right of *D*). *Left* image depiction of immunostaining shows a negative control (denoted “Neg” in *D*). (*F*) Correlation plot between PTOA local pain (in Fig. 4*A*) and LOR index (composed of mineral meniscus and osteophyte grading). PTOA *Lef1^fl/fl^* mice are represented as orange diamonds and *ATC Lef1^fl/fl^* mice, represented as green diamonds. The data were subjected to Pearson’s correlation (*r* = −0.53, *P* = 0.049), wherein *r* value closer to 1 indicates a good fit to linear regression. (*G*) Differentially expressed genes are presented as the Z-score value based on LFC classified into *NF-κB* signaling, *WNT/TGF/BMP* pathways, and Pain Signaling, according to DAVID annotation. Green panels are up-regulated genes; red panels are down-regulated genes, based on *SI Appendix*, SI_7, (*n* = 3). (*H*) STRING network analysis of the most networked DNA-binding transcription factors (TFs) and receptors in *ATC Lef1^fl/fl^* chondrocytes. (*I*) Table of the most-networked DNA-binding TFs based on node connections. (*J*) Potential partners of *Sox9* (i.e., most-networked TF) based on node scores with *Fgfr2/3* and *Lgr6* receptors in red font, in ascending order of node scores.

Similar to the reduced tibial osteophytes in PTOA *ATC Lef1^fl/fl^* mice, the lateral compartment was significantly reduced for meniscal mineralization vs. *Lef1^fl/fl^* (Fig. 5 *A* and *B*), with similar meniscal mineralization of the medial compartment for shams (*SI Appendix*, SI_15*F*). Closer analysis of the lateral meniscal tissue revealed Mmp13 was reduced in the PTOA *ATC Lef1^fl/fl^* mice compared to *Lef1^fl/fl^* (Fig. 5*C*). Consistently, *ATC Lef1^fl/fl^* also exhibited reduced staining for Col1a1 compared to *Lef1^fl/fl^* (Fig. 5*D*), for both “external” lateral menisci (i.e., tip of the lateral menisci; “LM”) and “internal” LM (scheme to the right of Fig. 5*D*; graphs in Fig. 5*E*), yet shams exhibited unchanged Cola1 staining levels (*SI Appendix*, SI_15*G*). These data are in line with reduced collagen type 1 staining of PTOA mice subjected to combination treatment (SRT1720 and CA074me; *SI Appendix*, SI_11*F*), cumulatively suggesting that Lef1 abation may reduce collagen 1 protein levels in the lateral menisci, following PTOA.

Next, we assessed whether there may be a correlation between pain sensitivity in PTOA mice compared to our LOR scores. *ATC Lef1^fl/fl^* mice displayed higher pain thresholds and lower LOR as compared to the *Lef1^fl/fl^* group (Fig. 5*F*), cumulatively providing a significant inverse correlation, in line with previous clinical reports (37).

Overall, our data support the idea that inflammation may prompt Lef1-mediated OA pathogenesis, while also perpetuating an inflammatory state. To further investigate the inflammatory effects of Lef1 ablation, we employed DAVID annotation and detected reduced NF-κB pathways (*SI Appendix*, SI_7: Table A and heatmap in Fig. 5*G*). Both DAVID pathways and STRING network analysis confirm reduced Toll-like receptor (*Tlr*)-2, -7, and -9 expression and related networks (*SI Appendix*, SI_7: Table A and SI_8: Table D and SI_5*D* STRING map). These data indicate that ablation of *Lef1* may contribute to reducing inflammatory pathways related to *NF-κB* and *Tlr* signaling, abrogating cartilage catabolism and tissue damage (17, 18, 38). Notably, *SI Appendix*, SI_5*D* illustrates the STRING map for down-regulation genes networks, exhibiting that *Tlr2* is highly networked under the down-regulated categories, known to be associated with OA-related pain (39–41), which is reduced in PTOA *ATC Lef1^fl/fl^* (Fig. 5*F*).

**Fig. 6.**
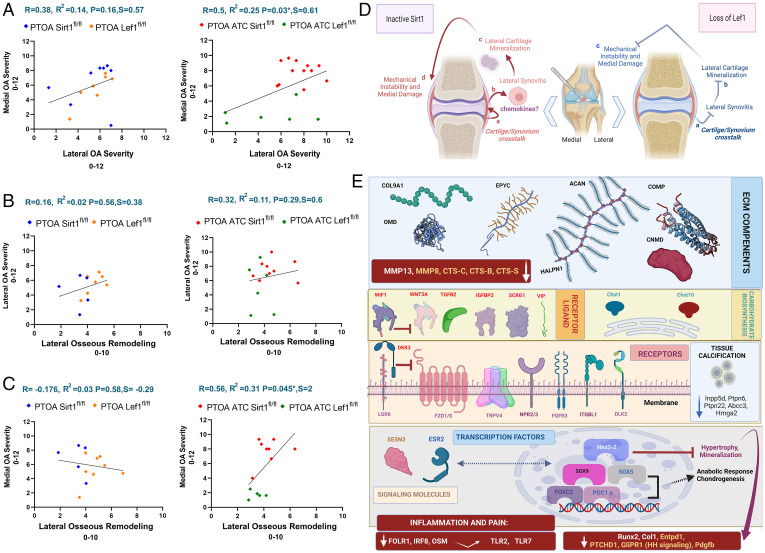
Correlation between joint compartments in controls (*Sirt1^fl/fl^* and *Lef1^fl/fl^*; blue and orange diamonds, respectively) and cartilage-specific transgenes (*ATC Sirt1^fl/fl^* and *ATC Lef1^fl/fl^*; red and green diamonds, respectively) display compartmental effects stemming from the lateral compartment. (*A*) Correlative comparisons of PTOA mice from the different strains (*Lef1^fl/fl^* vs. *Sirt1^fl/fl^* and *ATC Sirt1^fl/fl^* vs. *ATC Lef1^fl/fl^*) for cumulative (0 to 12) medial OA severity vs. cumulative (0 to 12) lateral OA severity. Sirt1 transgene OA severity data were taken from Batshon et al. (26). (*B*) Correlative comparisons of PTOA mice from the different strains (*Lef1^fl/fl^* vs. *Sirt1^fl/fl^* and *ATC Sirt1^fl/fl^* vs. *ATC Lef1^fl/fl^*) for cumulative (0 to 12) lateral OA severity vs. lateral osseous remodeling (LOR, 0 to 10). (*C*) Correlative comparisons of PTOA mice from the different strains (*Lef1^fl/fl^* vs. *Sirt1^fl/fl^* and *ATC Sirt1^fl/fl^* vs. *ATC Lef1^fl/fl^*) for cumulative (0 to 12) medial OA severity vs. LOR. Pearson’s correlation was assessed for mice samples, assuming a confidence level greater than 95% (*P* < 0.05) to be significant. Notably, Pearson's correlation (*r*) that is closer to 1 indicates a good fit to linear regression, while values closer to 0 indicate weak fit to linear regression. Regression (*r*^2^) indicates the variation around the linear regression line. Above each graph the *R*, *R*^2^, and slope, *P* value, appear for the correlation. (*D*) Graphical scheme illustrating changes in the joint compartments as OA progresses, in PTOA cartilage-specific Sirt1 ablated (*Left*; *a*–*d* steps) or cartilage-specific Lef1 ablated (*Right*; *a*–*c* steps) exhibit opposing structural changes predominantly in the lateral joint compartment. (*E*) Illustration of Lef1-ablated chondrocytes based on a selection of *SI Appendix*, SI_9 differentially up-regulated or down-regulated genes, which were manually mined via literary references for cartilage biology, tissue mineralization, and osteoarthritis. Up-regulated ECM components: *Col9a1*, *Acan*, *Hapln1*, Epyc, COMP, *OMD*, and *Cnmd*. Up-regulated enzymes contributing to carbohydrate biosynthesis include: *Chst-1* and *-10*. Down-regulated genes (red boxes; white font for PCR detected; yellow font RNA-Seq detected) include matrix-degrading enzymes (i.e., *Mmp-13*, *-8*, *Cts-C*, *-B*, and *-S*). Up-regulated ligands and growth factors include: *Wif1*, *Wnt5a*, *Tgfb2*, *Igfbp2*, and Scrg1. Up-regulated receptors include *Lgr6*, *Dkk3*, *Fzd-1*, and* -8*, *Trpv4*, *Npr-2–3*, *Fgfr3*, *Itgbl1*, and *Dlk2*. Up-regulated cellular signaling molecules include Sesn3 and *Esr2*, which can act as a transcription factor (arrow). Up-regulated transcription factors include: *Nkx3-2*, *SOX-5*, *-9*, *PGC1α*, and *FoxC2*, which may promote cartilage anabolic gene expression. Red box below exhibits reduced genes linked to joint inflammation and pain as *Irf8*, *Folr1*, *OSM*, and Tlr-*2* and *-7*. Additionally, genes related to hypertrophy and mineralization include *Runx2*, *Col1a1*, *Entpd1*, *Pdgfb*, and *hedgehog (HH)* signaling genes (i.e., *Ptchd* and *GliPR1*). Moreover, down-regulated genes that are linked to noncartilaginous tissue mineralization include: *Inpp5d*, *Ptpn6* and *-22*, *Abcc3*, and *Hmga2*.

Additional transcriptomic DAVID and STRING network analysis revealed that WNT5a, Frizzle receptors 1, 8, and 9 are increased in the Lef1-ablated chondrocytes (*SI Appendix*, SI_7: Table B, Fig. 5*G*, and *SI Appendix*, SI_8: Table B and SI_5*B*, STRING map, respectively). Contrary to previous reports (13, 42), here the overexpression of WNT5a did not result in OA phenotypes, possibly due to the overexpression of WNT inhibitors, such as *Dkk3* and *Wif1* (Dataset SI_9: up-regulated), detected in *Lef1*-ablated chondrocytes (Fig. 6*E*, scheme). Finally, STRING analysis of up-regulated DNA binding factors (*SI Appendix*, SI_8: Table A and Fig. 5*H*) revealed that *Sox9* is the most networked transcription factor among these up-regulated binding factors (Fig. 5 *H* and *I*), linked to other transcription factors (i.e., ascending score; *Snai2*, *Nkx6-1*, *Msx2*, *Nkx3-2*, *Ppargcgc1α*, or *PGC1α*) (Fig. 5 *H* and *J*), but also associated with receptors *Fgfr2/3* (Fig. 5 *H* and *J*), the latter shown to induce *Sox9* expression (43).

### Joint Compartmental Correlations between Transgenes Reveal That the Medial Articular Damage Is Affected by the Extent of Lateral Osseous Remodeling.

So far, we conclude that *ATC Sirt1^fl/fl^* mice show severe OA structural alteration, while *ATC Lef1^fl/fl^* mice developed relatively mild OA, denoting possible extremes of the same PTOA model. Here we attempted to underpin OA changes between the various joint compartments among these genetic models. As such, we plotted the control PTOA mice (i.e., *Sirt1^fl/fl^* and *Lef1^fl/fl^*) and cartilage-specific KO (i.e., *ATC Sirt1^fl/fl^* and *ATC Lef1^fl/fl^*) on the same graphs for the various measures, wherein OA measures from Sirt1 transgenes are taken from Batshon et al. (26).

Initially, we correlated between cumulative OA severities of the medial articular compartments (0 to 12) vs. the lateral articular compartments (Fig. 6*A*), for *Sirt1* transgenes (26). Both controls (i.e., *Sirt1^fl/fl^* and *Lef1^fl/fl^*) show a weak correlation (*P* = 0.16) between these compartments, while cartilage-specific KO (i.e., *ATC Sirt1^fl/fl^* or *ATC Lef1^fl/fl^*) show a stronger and statistically significant (*P* = 0.03) correlation, highlighting the two possible extremes for PTOA joint–related outcomes.

Next, we assessed the possible correlation of either lateral articular compartment (Fig. 6*B*) or medial articular compartment (Fig. 6*C*) with LOR (0 to 10 score, as in *Materials and Methods*). Lateral articular damage did not exhibit strong or statistically significant correlations with LOR for both flox controls (Fig. 6 *B*, *Left* graph) or cartilage-specific KO (Fig. 6 *B*, *Right* graph), indicating that these structural changes in the lateral compartment are loosely dependent. Similarly, examining the WT strains for medial articular damage vs. LOR (Fig. 6 *C*, *Left*) exhibited weak and insignificant correlation. This trend is reversed in the cartilage KO mice (Fig. 6 *C*, *Right*). Specifically, *ATC Sirt1^fl/fl^* and *ATC Lef1^fl/fl^* are separately clustered and display the strongest correlation, which is statistically significant (*R*^2^ = 0.31, *P* = 0.045) to show dependency between the medial articular damage vs. LOR, in the PTOA model for these transgenes. These data may indicate that reduced LOR may better maintain medial (not lateral) articular cartilage in PTOA (Fig. 6 *D*, *Right* illustration for Lef1 transgenes, steps *a* and *b*). Conversely, enhanced lateral synovitis (Fig. 6 *D*, *Left* illustration for Sirt1 transgenes, steps *a*–*c*) may potentiate LOR and medial articular damage following PTOA. The potential cross-talk between cartilage tissue devoid of *Sirt1*, may prompt augmented chemokine and proinflammatory signals to elicit synovitis as detected in *ATC Sirt1^fl/fl^* (*SI Appendix*, SI_12 *A* and *B*) to potentially harbor LOR phenotype (Fig. 6 *D*, *Left* illustration, steps *a*–*d*). Of note, *Sirt1* was reported to be highly expressed in the lateral joint compartment (44), cumulatively supporting the idea that molecular cartilage–related secretome of the lateral compartment may lead to medial damage, which is highly prevalent in OA.

Fig. 6*E* provides a molecular summary of the selected up-regulated and down-regulated genes, based on the Dataset SI_9 transcriptomic and PCR analysis identified in *Lef1*-ablated chondrocytes. Particularly, gene targets in SI9 that were manually data mined according to their relation to OA, cartilage biology or tissue mineralization, are presented in the scheme of Fig. 6*E*. The molecular signature in *Lef1*-ablated chondrocytes includes a host of up-regulated ECM components (i.e., *collagen type -9a1*, *Acan*, *Halpn1*, *Epyc*, *Comp*, *Omd*, and *Cnmd*), and enzymes participating in carbohydrate biosynthesis (*Chst-1* and *-10*). Matrix-degrading enzymes that were down-regulated (i.e., *Mmp-13*, *-8*, *Cts-C*, *Cts-B*, and *Cts-S*), indicating a gross anabolic response in the Lef1-ablated chondrocytes. While some WNT pathway receptors and ligands were up-regulated (*Lgr6*, *WNT5a*, *Frzl-1*, and *-8*), other inhibitory WNT constituents also appeared up-regulated (*Wif1* and *Dkk3*), possibly interfering with WNT signaling transmission.

Additionally, we detected up-regulated growth factors with protective roles toward OA (i.e., *Tgfb2*, *Igfbp2*, *Vip*, and Scrg1), up-regulated receptors (*Trpv4*, *Npr2*, *Npr3*, *Fgfr3*, *Itgbl1*, and *Dlk2*), signaling molecules (*Sesn3 and Esr2*), and transcription factors (*Nkx3-2*, *Sox9*, *Sox5*, *PGC1α*, and *FoxC2*). Notably, Nkx3-2 was reported to inhibit hypertrophy (Dataset SI_9), possibly leading to reduced bone-related markers as seen in our PCR (i.e., *Runx2* and *Col1a1*) and transcriptomic data (*Entpd1* and *Pdgfb*). Furthermore, reduced hedgehog (*HH*) signaling genes *Ptchd* and *GliPR1* were also observed and may also contribute to an abrogated mineralization (Dataset SI_9). Finally, red boxes in Fig. 6*E* exhibit reduced genes linked to joint inflammation and pain (i.e., *Irf8*, *Folr1*, *Osm*, and *Tlr 2/7*) (Dataset SI_9).

## Discussion

In the context of OA pathogenesis, the extent of cartilage mineralization may potentiate pain and alter load bearing of a joint (3–5). In cartilage, WNT signaling is largely associated with chondrocyte catabolism, hypertrophy, and mineralization, which may contribute to structural alterations of the joint, as osteophyte formation or mineralization of the meniscus (5, 45–48). However, agonists of the WNT pathway were reported to either prevent OA (12, 13) or adversely exacerbate OA (10, 11, 13). Similarly, in models of fracture repair, WNT activation promoted callus hypertrophy and repair (48, 49); however, attempting to enhance WNT signaling by sclerostin-neutralizing antibodies did not expedite repair at advanced stages of fracture callus remodeling (50). Hence the role of WNT signaling in cartilage catabolism and mineralization remains elusive, indicating that this pathway is adversely regulated in chondrocytes and cartilage.

Previous work implied that Sirt1 may activate WNT signaling, by β-catenin deacetylation to promote stem cell differentiation (30), while we reported that chondrocyte subjected to inflammatory processes displayed inactive Sirt1 (51) accompanied by increased Lef1 levels and cartilage catabolism (18). Here we used a PTOA model for two transgenes (either ablated for *Lef1* or *Sirt1* in cartilage) to examine a potential link between Sirt1 and Lef1. Surprisingly, the *Sirt1*-ablated PTOA mice exhibited lateral synovitis and LOR, which was not observed in the *Lef1*-ablated mice. As such, it appeared that cross-talk between cartilage and bone or synovium are largely instigated in the lateral compartment. One explanation for this compartmental effect of Sirt1 is its increased expression in the lateral compartment, as previously reported (44). Chondrocytes ablated for *Sirt1* highly express genes encoding secreted factors that may contribute to synovitis (i.e., *Cxcl9*, *Cilp*, *Gbp2b*, and *Tnc*) (34–36, 52), which were less detected in Lef1-ablated chondrocytes. Overall, the results support the idea that lack of Sirt1 could amplify inflammation following PTOA, while alternatively, PTOA-related inflammation may independently drive *Sirt1* cleavage to potentiate its proinflammatory effects during OA pathogenesis. Supporting the latter notion is the reduced OA severity detected in PTOA mice treated with a combination treatment activating *Sirt1* and simultaneously blocking its cleavage via *cathepsin B* inhibitor.

However, *Lef1*-ablated mice exhibited increased ECM biosynthesis, which indicated a strong anabolic effect (Fig. 6*E* and Dataset SI_9). Moreover, Lef1-ablated mice exhibited reduced cartilage mineralization, possibly a result of impaired *Entpd1* (e.g., *NTPDase1*), which is responsible for ATP hydrolyses (53, 54), and *HH* signaling genes (i.e., *Ptchd* and *GliPR1*) (55, 56) (Fig. 6*E*), as detected in our transcriptomic analysis. An additional down-regulated gene that may regulate tissue mineralization is *Trpv2*, which was recently reported to promote ectopic cartilage mineralization (57). Additional genes that were down-regulated in Lef1 nulls and may contribute to cartilage mineralization included: *Inpp5d*, *Ptpn6*, *Ptpn22*, *Atcc3*, *Hmga2*, and *Pdgfb* (Dataset SI_9). In particular, *Inpp5d* knockout was associated with an osteoporotic phenotype (58); polymorphism of *Ptpn6* caused its impaired function and associated with low bone mass (59); polymorphism of *Ptpn22* was linked to atherosclerotic stroke and arterial calcification (60, 61); *Abcc3* expression was associated with risk to atherosclerotic plaque (62); *Hmga2* was detected in calcified osteochondromas (63); and *Pdgfb* KO exhibited reduced brain calcification (64). Conversely, *OPG* was up-regulated in the Lef1-null chondrocytes and previously shown to prevent vascular calcification (65). Some of these genes are known to affect mineralization of noncartilaginous tissues, yet may additionally modulate cartilage mineralization, depending on *Lef1* and/or the inflammatory context of the joint.

On a physiological level, as mentioned previously, this Sirt1/Lef1 molecular axis appears to impact cartilage catabolism and mineralization, especially in the lateral joint compartment. By reducing mineralization of the lateral joint compartment, we may effectively prevent damage to the medial compartment by maintaining the mechanical stability and load bearing of the joint. In line with this notion, previous reports show that matrix composition and stiffness can evoke OA via shifted mechanotransduction (66). Interestingly, ectopic mineralization is detected radiographically and often termed chondrocalcinosis. Accordingly, detection of chondrocalcinosis may potentially and locally guide drug administration in a compartmentalized manner, to ultimately achieve better disease modification effects (67).

In summary, we provide a molecular axis linking inflammation to cartilage mineralization following posttraumatic OA induction. This axis caused significant and opposing structural changes in the lateral compartment, leading to medial articular damage, which is characteristic of OA. Therefore, future attempts to employ imaging for the detection of lateral cartilaginous mineralization or chondrocalcinosis, may support localized treatments of the affected joint compartments, to prevent OA damage and disability.

## Supplementary Material

Supplementary File

Supplementary File

## Data Availability

The datasets generated during and/or analyzed during the current study are available in the article and *SI Appendix*. Raw transcriptomic data are deposited in the Gene Expression Omnibus repository (accession number GSE200522).
