# Supplementary material for: Lef1 ablation alleviates cartilage mineralization following posttraumatic osteoarthritis induction
Source: Proc Natl Acad Sci U S A. 2022 May 20;119(21):e2116855119. doi: 10.1073/pnas.2116855119 (PMC9173807; doi:10.1073/pnas.2116855119)
Supplement: Supplementary File [file pnas.2116855119.sapp.pdf]

MATERIALS AND METHODS:

Human Cell Cultures

Human chondrocytes were isolated from end-stage OA patients who underwent total knee arthroplasty, (n=25, mean age 71 years, mean body mass index 30.5 kg/m2). Written informed consent was obtained from all patients prior to the procedure. The study protocol included clinically established end-stage OA, based on Kellgren and Lawrence (KL) score of 3-4, according to radiographic evidence of the affected knee joint. The full study protocol was approved by the Institutional Ethics committee of Hadassah Medical Center (Institutional approval #04880-09-HMO) [1]. Monolayer cultures were maintained in Dulbecco's Modified Eagle's Medium (DMEM) supplemented with 10% fetal bovine serum (FBS), 50 U/ml penicillin and 50 µg/mL streptomycin (Biological Industries, Kibbutz BeitHaemek, Israel). Cells were treated with pro-inflammatory cytokines IL1β (5ng/mL; Peprotec, Rehovot, Israel) and TNF-α (50ng/mL; Peprotec Rehovot Israel) in defined serum-free media (BIO-MPM 1; Biological Industries, Kibbutz Beit Haemek, Israel).

Mice Experimental Procedures

Mice related experimental procedures were carried out in accordance with NIH Committees for animal use and care (ARAC guidelines) and based on Association for Assessment and Accreditation of Laboratory Animal Care International (AAALAC) guidelines. The Hebrew University Institutional Animal Care and Use Committee approved the study's protocols (MD-12-13383-4; MD-14-14172-2; 1-15660-18). Mice were subjected to 12-h light/dark cycles and received food and water as libitum.

Cartilage-specific Sirt1-knockout mice (*ATC Sirt1*$^{fl/fl}$), were generated by breeding *Sirt1*$^{fl/fl}$ (Jackson Laboratory, #008041) with *ATC* transgenic mice (provided by Prof. Veronique Lefebvre, CHOP Research Institute, PA), as previously described [2]. Similarly, *ATC* transgenic

mice were crossed with *Lef1*$^{fl/fl}$ (provided by Prof. Hai-Hui Xue, University of Iowa, [3]) to generate cartilage-specific Lef1-knockout mice (*ATC Lef1*$^{fl/fl}$). Notably, *ATC* transgenic mice express an Agc1 enhancer-driven, tetracycline-inducible Cre (ATC) transgene [4], which we induced in three months old adult mice, two weeks prior to post-traumatic OA (PTOA) procedures, or in pregnant dams between E11.5 to E17.5 gestational days, by adding doxycycline (0.8 mg/400 mL; Biobasic, Canada, cat# 24390-14-5) to drinking water.

PTOA experiments were carried out by *surgical* destabilization of the medial meniscus (DMM) or a sham (i.e. exposed and unoperated joint) procedures, as previously described [5]. Mice were euthanized by cervical dislocation following anesthesia (Ketamine and Xylazine 200 mg/kg). Local Joint Pain measurements were carried out using a Pressure Applicator measurement device (PAM, Ugo Basile, Italy) prior to euthanizing the mice, 8 weeks post-procedure. During PAM procedure mice are restrained by the operator, which simultaneously applies pressure to the mouse joint using a special force sensor on the operators' thumb. The force applied, which elicits the animal response (i.e. limb withdrawal), is automatically recorded. Reduced gram forces in PTOA, indicates that less pressure was required for limb withdrawal, to imply local joint sensitization to pain. Each measurement consists of triplicate of measurements for each mouse with 1-minute break between.

C57BL/6 mice (Jackson lab) WT mice at three months old were subjected to PTOA, as previously described. Following 4 weeks after surgical induction, mice were injected intra-articularly (IA) for the following 4 weeks, twice a week, as follows: Vehicle (20 %v/v DMSO in PBS), *Sirt1* activator (i.e. SRT1720; 81μM in 5μL; ApexBio, cat#A8239); or Cathepsin B inhibitor (CA074me; 600 μM, in 5μL; BioVision, cat#2772); or combination of *Sirt1* activator 1 and Cathepsin B inhibitor (concentrations as indicated above, overall 10 μL volume). After 8 weeks

PTOA, the mice were euthanized by cervical dislocation following anesthesia (ketamine and xylazine 200 mg/kg). Joints were collected for histological analysis, as detailed below.

Mouse Embryonic E17 Skeletal Phenotyping and Chondrocyte Cultures

Adult three months old mice were anesthetized and sacrificed, to harvest tibiofemoral joints for articular chondrocyte culture. Costal chondrocytes were extracted from E17 embryos obtained from pregnant dams subjected to anesthesia (Ketamine and Xylazine 400 mg/kg), and cervical dislocation. Extracted joint tissues, and dissected ribcage tissues were incubated with collagenase solution (3 mg/mL in DMEM; cat# LS004177, Worthington Biochemical Corporation, NJ) at 37°C for 1h, following a single wash step with PBS. Next an overnight incubation with 0.5 mg/mL collagenase in DMEM, was carried out at 37°C, following tissue filtration in a 40μm-mesh cell strainer, subsequent to cell culture.

For skeletal phenotyping of E17 embryos, pregnant dams were anesthetized, as indicated previously. Extracted embryos were then immersed in 70% ethanol and soft tissue was removed using surgical scissors. Embryos were then fixed in 95% ethanol at room temperature (RT) and transferred to 100% acetone overnight at RT. Next, embryos were stained with 0.03% Alcian Blue (cat# A5268-25G, Sigma-Aldrich) in 80% ethanol and acetic acid overnight at RT, followed by a distaining step carried out by 3 washes in 70% ethanol and overnight incubation in 95% ethanol RT, in rotation. To remove residual soft tissue, embryos were transferred to 1% KOH solution for 1 hour at RT, followed by staining with 0.005% Alizarin Red (cat# A5533-25G, Sigma-Aldrich) in 1% KOH, for 3-4 hours at RT in rotation. Following staining, the solution was replaced with 1:1 glycerol: 1% KOH solution at RT, until soft tissue was removed, typically within 48 hours. Finally, embryos were transferred to 100% glycerol for long-term storage at RT.

Chromatin Immunoprecipitation (ChIP)

To carry out ChIP assays, human or mouse costal chondrocytes were cultured and treated, as indicated. Following treatment, cultures were washed with PBS and cross-linked with 1% paraformaldehyde (cat# BN15710, Electron Microscopy Sciences) for 15 min, which was stopped by adding 0.125M glycine at RT. Cells were then lysed in ice cold lysis buffer (10 mM EDTA, 50 mM TrisHCl, pH 8.0, 1% sodium dodecyl sulfate (SDS), and supplemented with Protease Inhibitor Cocktail (PI 1:50 cat #C01010073, Diagenode, Belgium), which additionally included 1:100 PMSF (cat# P7626, Sigma-Aldrich); 1:200 Sodium Butyrate (cat#B5887, Sigma-Aldrich), 1:1000 TSA (cat#T8552, Sigma-Aldrich); 1:500 ALLN (cat# 208719, Sigma-Aldrich) and 1:500 NAM (cat#72340, Sigma-Aldrich). Following lysis, the cell extracts were sonicated (Vibra Cell TM, Sonics, CA) at 50 cycles of 95% amplitude for 30 sec, followed by a 45 sec incubation on ice, based on Bar Oz *et al* [6]. Samples were then processed using the Low Cell ChIP kit (cat #C01010073, Diagenode, Belgium) and immunoprecipitated with SIRT1 (C-term, Bethyl Laboratories, #A300-688A), SIRT1 (N-term, Millipore, #07-131), a-Acetyl-Histone H4 (Lys16) (cat# 06-762, Millipore, MA) or LEF1 (cat# 17-604 Millipore, MA) antibodies. Following DNA isolation, quantitative real-time polymerase chain reaction (qPCR) was carried out for 11 tandem primer pairs (tile ChIP), based on a previous report by Li *et al*., 2006 [7], of the regulatory sites on the LEF1 gene, encoding the full length LEF1 transcript in humans (**SI_2:table a**)**.** ChIP was additionally carried out for the 3′ region of genomic mouse MMP13 (NG_021404) and c-myc gene, which carries putative LEF1 DNA binding sites **(SI_2:table a).**

Immunoblot analysis

Crude protein extracts were generated, as previously described [7] and run, on standard 10% SDS polyacrylamide gel electrophoresis (PAGE), which was transferred to polyvinylidene difluoride (PVDF) membranes for immunoblotting, as previously described [6, 8]. Primary antibodies used were LEF1 (Millipore, MA; cat# 17-604), β-actin antibody (SC-47778, Santa

Cruz, CA) and GAPDH (#CB1001, Millipore), while secondary antibodies were alkaline-phosphatase (AP)-conjugated anti-mouse (Sigma-Aldrich, cat#A3562) or anti-rabbit (Sigma-Aldrich, cat#A3687). The blots were developed with BCIP/NBT and scanned in high resolution to determine band intensity via ImageJ software. Each band was normalized to the corresponding housekeeping protein appearing on the blot. Relative band intensity was presented in arbitrary units (A.U.) adjacent to the representative immunoblot.

Immunohistochemistry,* immunofluorescence *and histological procedures

Immunohistochemistry (IHC) for harvested murine joints was carried out following 2 days of tissue fixation in 4% paraformaldehyde in PBS and 21 days of decalcification with 10% EDTA (Sigma-Aldrich) in double distilled water (DDW) at (pH=7.4). Samples were then dehydrated using a graded series of ethanol washes, embedded in paraffin, and sectioned to 7-μm slices. Sequential coronal sections were obtained until reaching the medial to lateral plane (approx. 1500μM in depth, based on the embedded sample orientation). Then 20 slides (2 sections per slide) at 7μm thickness each, were obtained for staining procedures. Sections were digested with 1 mg/mL hyaluronidase (Sigma-Aldrich, cat#H3506) in PBS at pH=6 for 1 h at 37° C and incubated overnight with primary antibodies; MMP13 (cat# ab39012, Abcam, UK), F4/80 CI:A3-1 (cat# MCA497GA, Biorad laboratories, CA); COL1A1 (cat# ab21286, Abcam). ZytoChem Plus HRP polymer conjugated anti-rabbit (cat#ZUC032, Zytomed Systems, Germany) was used as a secondary antibody followed by DAB substrate kit (cat#550880, BD Pharmingen, CA) for color development. Negative controls were incubated with secondary antibody alone and counterstained with hematoxylin. IHC sections stained with MMP13, F4/80 CI:A3-1 and COL1A1 were quantified by ImageJ IHC_Toolbox.jar plugin per standard analyzed area to detect staining intensity. COL1A1 staining was assessed via two standard boxes created in

ImageJ software for external and internal meniscal sites of the lateral compartment (see **Fig. 5**).

For immunofluorescence, sections were rehydrated with decreasing concentrations of ethanol, incubated for 1 h with blocking solution containing 0.1% BSA, 1:10 FBS and 1:50 Triton x100 in PBS, room temperature (RT). Next, sections were incubated with rabbit Anti-LEF1 antibody (cat# ab137872; Abcam) overnight at 4 degrees Celsius (1:150 in blocking solution). The following day, sections were incubated with Alexa flour 568 anti-rabbit (1:1000 in blocking solution, #cat A-11011, UK, Abcam), for 2 h at RT. Finally, sections were incubated with DAPI (final 5ng/μL in DDW) for 10min at RT, washed four times with PBS and mounted (IMMU-MOUNT, MA, Thermo Fisher). Sections were visualized under a Ti-Eclipse Nikon system with an Andor Zyla nsc05537 camera (Japan, Nikon). Captured depictions were analyzed for coappearance of DAPI/LEF1 intensity and % positive nuclei, using NIS-elements BR software (Japan, Nikon).

To determine OA histopathology in murine joints, sections were stained with 0.5% Safranin O (cat# 1.15948, Millipore) and 0.1% Fast green (cat# 1.04022, Millipore) after using Wiegert's Iron Hematoxylin (cat# 1.15973, Merck) in DDW. OA histopathology scoring was carried out based on the 0-6 scale for OA severity (6 denoting the most severe case), according to a Glasson *et al.* 2010 method [9]. For OARSI severity scoring we obtained 1-3 sequential (80μm-spaced) coronal sections, which were stained and graded by three blinded graders, according to the scoring criteria of Glasson et al. 2010. Average scores of graders are presented in graphs. Notably, OA severity between females vs. males for all genotypes (i.e., *ATC Sirt1*$^{fl/fl}$, *Sirt1*$^{fl/fl}$, *ATC Lef1*$^{fl/fl}$ and *Lef1*$^{fl/fl}$; sham or PTOA) were insignificant (**SI_2: table b; table c,** respectively**).**

Osteophyte formation was monitored on a 0-3 scale (3 denoting the most severe case), based on the method by Kamekura S*., et al*.[10]. Additionally, the degree of synovitis was determined based on synovial thickness and appearance of F4/80 positive cells within the synovial membrane area. Briefly, synovial lining was stained by H&E staining (Mayer's Hematoxylin, cat# 1100, Kaltek; Eosin Y, cat# 3801601E, Leica Biosystems) and the membrane region was extracted manually from each joint image, to be subsequently uploaded to ImageJ software. Using ImageJ processing tools, the number of colored pixels were converted to cm^2, to indicate the thickness of the selected synovial membrane area. Secondly, IHC of F4/80+ (brown) macrophages was carried out and quantified by ImageJ:IHC_Toolbox.jar plugin, per analyzed area for F4/80 positive cells.

Micro computed tomography (μCT) Analysis

Harvested joints were fixed in 4% paraformaldehyde solution for 48h and subsequently stored in 70% EtOH, for μCT assessments of calcified tissue using Skyscan 1174 (Bruker, Belgium; 50 kV,0.25 aluminum filter, 4000 ms exposure time, 6.4 μm ,0.4° rotation angle, 7.95 μm pixel size). Scans were reconstructed in NRecon software (Bruker, Belgium). The scans were converted to longitudinal sections and the area of interest was selected as the tibial subchondral bone plate and mineralized meniscus using CTan software (Bruker, Belgium). Compartments were manually segmented into medial and lateral regions of interest (ROIs). Three-dimensional (3D) analysis was assessed to quantify bone volume of mineralized meniscal tissue and percent of bone volume/tissue volume (%BV/TV) and cortical thickness of tibial subchondral cortical bone plate using CTan software. The data obtained was analyzed and compared between PTOA *ATC* $Sirt1^{fl/fl}$ *and* $Sirt1^{fl/fl}$*;* or PTOA *ATC* $Lef1^{fl/fl}$ *and* $Lef1^{fl/fl}$ mice, as well as respective sham controls. 3D images were produced with Dragonfly software (ORS, Montreal, Canada).

RNA isolation and qPCR analysis

Total RNA was extracted from cells using the RNeasy kit (Qiagen, Valencia, CA, USA). Oligo dT was used as the primer in the reverse-transcription reaction. Quantitative real-time PCR (qPCR) reactions were performed with 10 ng of cDNA and Syber Green mix (BioRad Laboratories, Hercules, CA, USA). Quantitative analyses were performed with iCycler software (Bio-Rad, Hercules, CA, USA). All RNA samples were treated with DNase I before the PCR reactions. Human and mouse primers used for gene expression and genotyping are in **SI_2 (table d).** Values were normalized to GAPDH, which remained unaffected by the experimental treatments.

RNAseq Data Acquisition

Mice costal chondrocytes derived from *Lef1* $^{fl/fl}$ (n=3), *ATC Lef1* $^{fl/fl}$ (n=3), and *ATC Sirt1* $^{fl/fl}$ (n=2) were extracted, cultured and plated to reach passage 2. Following 24h doxycycline treatment, total RNA was isolated and assessed for quality according to EMBL guidelines using a TapeStation Analysis Software A.02.02 (SR1). RNA integrity numbers (RIN) for all samples were > 9.3. Plate-based RNA-Seq with poly(A) selection sample prep was performed using the KAPA Stranded RNASeq Library Preparation Kit (Illumina® platforms), product codes KK8400 and KK8401. For quality control of RNA yield and library synthesis products, the RNA ScreenTape and D1000 ScreenTape kits (both from Agilent Technologies), Qubit® RNA HS Assay kit, and Qubit® DNA HS Assay kit (both from Invitrogen) were used for each specific step. mRNA libraries were prepared from 1 µg RNA using the KAPA Stranded mRNA-Seq Kit, with mRNA Capture Beads (kapabiosystems, KK8421, https://www.kapabiosystems.com/). Each library was eluted in 20 µl elution buffer, pooled libraries was adjusted to 10 mM. The multiplexed sample pool (1.6 pM including PhiX 1%) was loaded on NextSeq 500/550 High Output v2 kit (75 cycles) cartridge, and loaded onto the NextSeq 500 System (Illumina, San

Diego, CA, USA), with 75 cycles and single-read sequencing conditions. Sequencing data sets have been deposited in the GEO database- accession number (**GSE200522**). Prior to sequence analysis, quality control of RNA-Seq samples was performed on the raw single-end reads using Fastqx to remove low-quality reads (average score less than 30). Reads that were less than 20 base pairs were discarded. In addition, all reads were cut at the ends, leaving the sequence between 14-54 base pair, due to low-quality base readings. Reads were then aligned to the *Mus Musculus* genome GRCm38 version with STAR [11], using standard presets except for intron size, which was set between 30 and 30000 bp (–alignIntronMin 30 and –alignIntronMax 30000). Greater than 10 million reads were mapped for each sample with uniquely mapping reads accounting for ~90% of total mapped reads in each sample. Uniquely mapping reads were count using HT-Seq [12]. Prior to analysis, PCA plot was validated to determine that datasets are sufficiently variable. Next, differential expression analysis was performed on *Lef1* $^{fl/fl}$ and *ATC Lef1* $^{fl/fl}$ samples using DESeq2 [13]. In general, a total of 918 differentially expressed genes based on having a 1-fold expression difference from their mean expression with adjusted p-value <0.1; comprising of 416 up-regulated and 502 down-regulated genes. DESeq2 on *ATC Lef1* $^{fl/fl}$ (n=3) and *ATC Sirt1* $^{fl/fl}$ (n=2) generated a total of 274 differentially expressed genes based on having a 1-fold expression difference from their mean expression with adjusted p-value <0.1; 178 up-regulated and 96 down-regulated genes. **SI_3A-C**, present PCA, Volcano plot and differentially expressed gene plot for all comparisons. An additional PCA plot (**SI_3D**) compares C57BL costal chondrocytes (E17.5, C57B6 background) from *Lef1*$^{fl/fl}$ with samples from adult articular chondrocytes of same C57BL6 background, as derived GEO databanks for 6-week post sham procedure (GSE143447; 14) and un-operated joint 4 weeks post-procedure (GSE175486, 15). The comparison between costal chondrocytes and articular chondrocytes established a significant variation on the PC1 axis, which is the

reason for our assessment of expression and staining profiles amongst both cell types, as indicated within the results section (**Fig.1; Fig. 3; SI_3; SI_12**). Differentially expressed genes were calculated by 1-fold expression difference or greater (Log2 fold expression) compared to the *Lef1*$^{fl/fl}$ control or *ATC Sirt1*$^{fl/fl}$ *compared to ATC Lef1* $^{fl/fl}$ values, with an adjusted p-value lower than 0.1, which were presented in tables and heatmaps according to classification categories of interest.

Functional annotations analysis was preformed to assign biological relevance of the differentially expressed gene sets, derived from DESeq2 analysis and subjected to DAVID Bioinformatic resources 6.8 [16]. Employing DAVID tool enabled the analysis of gene clusters contributing to particular biological processes, which display differential amongst the *Lef1* $^{fl/fl}$, and *ATC Lef1* $^{fl/fl}$. Next, DAVID datasets were filtered for GO enrichment pathways, which were plotted in a Venn diagram for upregulated and downregulated GO enrichment gene sets (**SI_4**), wherein 5 categories were common and omitted from the next steps of analysis. As such, enrichment categories were plotted in GO classification (i.e. Molecular function, Biological process, Cellular component), with a total of 25 downregulated sets and, 20 upregulated sets **(SI_4**). Of those unique pathways, we identified common functional processes which were upregulated (i.e. WNT signaling, DNA binding and signaling, carbohydrate processing) or down regulated (i.e. Inflammation), separately. To identify key interactions and regulatory molecules from those processes, we employed STRING network analysis (**SI_5**; version 11.5 https://string-db.org/ [17]) with a common threshold of 0.4. Central nodes (Hub nodes) were identified as such by using cytoHubba (version 0.1, [18]) plugin in the Cytoscape software (version 3.8.2 [19]) based on the Degree topological analysis method. Relevant tables are presented in **SI_6-9**. For *ATC Sirt1*$^{fl/fl}$ *vs ATC Lef1*$^{fl/fl}$ comparisons (**SI_3E, SI_3F**), we employed

DAVID datasets, which were filtered for GO enrichment cluster related to "Extracellular Region" and plotted based on the 10 most differentially expressed genes (**Fig. 4G, SI_10**).

Statistical analysis

Each experiment was repeated at least three times ($n \geq 3$) and the average and standard deviation was calculated per group, after removing statistically significant outliers, as determined by GraphPad prism software. All the data were analysed for non-parametric Kruskal-Wallis and post-hoc Dunn's test to assess statistical significance within a group of treatments, assuming a $p<0.05$. After confirming significance of Kruskal-Wallis test, we employed Mann-Whitney test to assess statistical significance between treatments. Statistical significance according to Mann-Whitney test is denoted with an asterisk (*) for $p<0.05$ above the relevant bar, or as indicated in figure legends. Pearson correlation was assessed for mice joint parameters, assuming a confidence level greater than 95% ($p<0.05$), to be significant. Notably, Pearson's correlation (r) that is closer to 1 indicates a good fit to linear regression, while values closer to 0 indicate weak fit to linear regression. Regression (r^2) indicates the variation around the linear regression line. The cumulative scores of OA severity per compartment (0-12) were plotted for the medial vs lateral correlations. Lateral Osseous Remodeling (LOR) was calculated by adding the cumulative score of osteophyte grading (0-6 for both tibia and femoral compartments) to the mineralized meniscal value (in $10nm^3$, as determined by microCT) only for the lateral compartment (0-4), on a total range of 0-10 for x-axis. Next, we comparted the LOR measure, with Pain thresholds (range 0-400gf) or medial or lateral OA severity (0-12), as indicated above. Graphs and statistical analysis were carried out in GraphPad Prism 9.0 software, while illustrations carried out via BioRender software.

REFERENCES (SI_1):

1) Oppenheimer, H., et al., *Set7/9 impacts COL2A1 expression through binding and repression of SirT1 histone deacetylation.* J Bone Miner Res, 2014. **29**(2): p. 348-60.
2) Dy, P., et al., *Sox9 Directs Hypertrophic Maturation and Blocks Osteoblast Differentiation of Growth Plate Chondrocytes.* Developmental Cell, 2012. **22**(3): p. 597-609.
3) Yu, S.Y., et al., *The TCF-1 and LEF-1 Transcription Factors Have Cooperative and Opposing Roles in T Cell Development and Malignancy.* Immunity, 2012. **37**(5): p. 813-826.
4) Batshon, G., et al., *Serum NT/CT SIRT1 ratio reflects early osteoarthritis and chondrosenescence.* Ann Rheum Dis, 2020. **79**(10): p. 1370-1380.
5) Glasson, S.S., T.J. Blanchet, and E.A. Morris, *The surgical destabilization of the medial meniscus (DMM) model of osteoarthritis in the 129/SvEv mouse.* Osteoarthritis and Cartilage, 2007. **15**(9): p. 1061-1069.
6) Bar Oz, M., et al., *Acetylation reduces SOX9 nuclear entry and ACAN gene transactivation in human chondrocytes.* Aging Cell, 2016. **15**(3): p. 499-508.
7) Li, T.W., et al., *Wnt activation and alternative promoter repression of LEF1 in colon cancer.* Mol Cell Biol, 2006. **26**(14): p. 5284-99.
8) Dvir-Ginzberg, M., et al., *Tumor necrosis factor alpha-mediated cleavage and inactivation of SirT1 in human osteoarthritic chondrocytes.* Arthritis Rheum, 2011. **63**(8): p. 2363-73.
9) Glasson, S.S., et al., *The OARSI histopathology initiative - recommendations for histological assessments of osteoarthritis in the mouse.* Osteoarthritis and Cartilage, 2010. **18**: p. S17-S23.
10) Kamekura, S., et al., *Osteoarthritis development in novel experimental mouse models induced by knee joint instability.* Osteoarthritis Cartilage, 2005. **13**(7): p. 632-41.

11) Dobin, A., et al., *STAR: ultrafast universal RNA-seq aligner.* Bioinformatics, 2013. **29**(1): p. 15-21.

12) Anders, S., P.T. Pyl, and W. Huber, *HTSeq-a Python framework to work with high-throughput sequencing data.* Bioinformatics, 2015. **31**(2): p. 166-169.

13) Love, M.I., W. Huber, and S. Anders, *Moderated estimation of fold change and dispersion for RNA-seq data with DESeq2.* Genome Biology, 2014. **15**(12).

14) Smeriglio, P. Grandi, F.C., Davala, S., Masarapu, V., Indelli, P.F., Goodman, S.B. and Bhutani, N, Inhibition of TET1 prevents the development of osteoarthritis and reveals the 5hmC landscape that orchestrates pathogenesis. Sci Transl Med., 2020. **12**(539).

15) Singh, P., Wang, M., Mukherjee, P., Lessard, S.G., Pannellini, T., Carballo, C.B., Rodeo, S.A., Goldring, M.B. and Otero, M, Transcriptomic and epigenomic analyses uncovered Lrrc15 as a contributing factor to cartilage damage in osteoarthritis. Sci Rep,. 2021 **11**(1):21107.

16) Dennis, G., et al., *DAVID: Database for annotation, visualization, and integrated discovery.* Genome Biology, 2003. **4**(9).

17) Szklarczyk, D., et al., *STRING v11: protein-protein association networks with increased coverage, supporting functional discovery in genome-wide experimental datasets.* Nucleic Acids Research, 2019. **47**(D1): p. D607-D613.

18) Chin, C.H., et al., *cytoHubba: identifying hub objects and sub-networks from complex interactome.* Bmc Systems Biology, 2014. **8**.

19) Shannon, P., et al., *Cytoscape: A software environment for integrated models of biomolecular interaction networks.* Genome Research, 2003. **13**(11): p. 2498-2504.

SI 2: Primer tables and sex related OA severity of transgenes: Composed of four tables (**a-d**): **Table a**: Primers of Lef1 Gene; **Table b**: OA severity between males and females of Sirt1 transgenes; OA severity between males and females of the same genotype and surgical procedure, based on Glasson et al., 2010 grading, and Mann Whitney U test (p>0.05). **Table c**: OA severity between males and females of Lef1 transgenes. OA severity between males and females of the same genotype and surgical procedure, based on Glasson et al., 2010 grading, and Mann Whitney U test (p>0.05); **Table d**: PCR and genotyping primers.

Table a: Primers of Lef1 Gene used for ChIP analysis

ChIP Primers Human LEF1 (P1 or P2)	Forward Primer	Reverse Primer
Primer 1	GAC TCG TCC TAC AGG ATC TGG	CTC GAG CTC GGC TGT GTG TGT G
Primer 2	TGT GTC GGC TCG AGC TCC GGG	GTT CTG GAG GGA GCA CGC CTC CCC AAA GTG
Primer 3	CAG AAC CGG CGT TCT CCG CGC GCA AAT CCC	CCT TCT GAG GAT CGC CCT CGT CCT TGA AGG
Primer 4	CCT TCA AGG ACG AGG GCG ATC CTC AGA AGG	AGC CTC TGC ACT TTG TTT TCC GTC CCA CGG
Primer 5	GGC TTA GGT GGA GAC GAG CAG AGC CAC AGG	TGC TCC GCG CTT TCT CTA GCC AGC ACC CAC
Primer 6	CCG ACA CTC CAC TGG TCA CCC TTG TTC CAC	CCA TTC TGT TCT CTG AAC GCA GGC CCC CAG
Primer 7	TAC CCG TTG AAT CCA GTT TGA GCG GGC TGG	CCT ACT ATG GCT GGC TGA GTG CAG GCT TAC
Primer 8	CGC TCA GGG CAG TTG CCT TAA GAC CGC ATC	XCC GTC ATC GGG GTG TTC TCT GGC CTT GTC
Primer 9	AGG GAC CCT TGG ATT TCA GGT GGG TTC CAG	CTT CCC TGC TCC TGG TTG ACA TAT TGT ATG
Primer 10	CTC AGC TAC AAG ATA GTT ACA CAG ATT AAC	TTC AAA CAG TTC TTC CAC TAC TGA CTG ATG
Primer 11	GAT AGG TAC CGG CTA AGC TAT TTA AGA GAA TA	TGT TCT CGG GAT GGG TGG AGA AAG
**ChIP Primers Mouse**		
Mmp13	CATGCCAACAAATTCCATATTG	CCAGCCACGCATAGTCATATAG
c-myc	CCC AAA AAA AGG CAC GGA A	TAT TGG AAA TGC GGT CAT GC

Table b: OA severity for males vs females of Sirt1 transgenes

Genotype/Surgery	Females<br>mean (MFC, MTP, LFC, LTP)	Males<br>mean (MFC, MTP, LFC, LTP)	Females vs Males<br>P-value (MFC, MTP, LFC, LTP)
**Sirt1$^{fl/fl}$ Sham**	(1.33, 0.83, 1.08, 1.16)	(1.61, 1.16, 2.11, 1.55)	(1, 0.72, 0.22, 0.85)
**Sirt1$^{fl/fl}$ DMM**	(2.25, 2.04, 2.20, 2)	(4.16, 4.16, 3, 3.5)	(0.06, 0.05, 0.30, 0.30)
**ATC Sirt1$^{fl/fl}$ Sham**	(1.5, 1.33, 2, 2.75)	(2.05, 1.61, 2.38, 2.33)	(0.72, 1, 0.85, 0.72)
**ATCSirt1$^{fl/fl}$ DMM**	(4, 3.33, 3.77, 3.33)	(4.40, 3.59, 3.70, 4.01)	(0.38, 0.51, 1, 0.516)

Table b: OA severity between males and females of the same genotype and surgical procedure, based on grading criteria of Glasson et al., 2010 grading, and Mann Whitney U test (p>0.05).

Table c: OA severity for males vs females of Lef11 transgenes

Genotype/Surgery	**Females** mean (MFC, MTP, LFC, LTP)	**Males** mean (MFC, MTP, LFC, LTP)	**Females vs Males** P-value (MFC, MTP, LFC, LTP)
Lef1$^{fl/fl}$ Sham	(1.81, 1.5, 1.31, 2.25)	(2.56, 1, 1.37, 1.93)	(0.30, 0.30, 0.88, 0.76)
Lef1$^{fl/fl}$ DMM	(2.62, 3.09, 3.03, 3.25)	(2.62, 3.29, 2.46, 2.81)	(1, 1, 0.55, 0.77)
ATC Lef1$^{fl/fl}$ Sham	(1.06, 2.62, 1.53, 1.40)	(1.15, 1.78, 1.15, 1.90)	(0.30, 0.48, 0.77, 0.30)
ATC Lef1$^{fl/fl}$ DMM	(0.75, 0.81, 3.04, 4.02)	(1.12, 2.91, 1.87, 2.40)	(0.06, 0.35, 0.22, 0.28)

Table c: OA severity between males and females of the same genotype and surgical procedure, based on grading criteria of Glasson et al., 2010 grading, and Mann Whitney U test ($p>0.05$).

Table d: PCR and genotyping primers

**Primers for Human RT-qPCR**	**Forward Primer**	**Reverse Primer**
GAPDH	TACTAGCGGTTTTACGGGCG	TCGAACAGGAGCAGAGAGCGA
LEF1	CAGTCATCCCGAAGAGGAAG	AGGGCTCCTGAGAGGTTTGT
**Primers for Mice RT-qPCR**	**Forward Primer**	**Reverse Primer**
Mmp13	GGAGCCCTGATGTTTCCCAT	GTCTTCATCGCCTGGACCATA
Col1A1	CGATGGATTCCCGTTCGAGT	CGATCTCGTTGGATCCCTGG
Lef1	TGAGTGCACGCTAAAGGAGA	ATAATTGTCTCGCGCTGACC
Runx2	GAG AGGTACCAGATGGGACT	CACTTGGGGAGGATTTGTGA
**Primers for Mice Genotyping**	**Forward Primer**	**Reverse Primer**
Sirt1$^{fl/fl}$	GGTTGACTTAGGTCTTGTCTG	CGTCCCTTGTAATGTTTCCC
Lef1$^{fl/fl}$	GCA-GAT-ATA-GAC-ACT-AGC-ACC	TCC-ACA-CAA-CTA-ACG-GCT-AC
ATC	TGAGGTTCGCAAGAACCTGATGGA	GCCGCATAACCAGTGAAACAGCAT

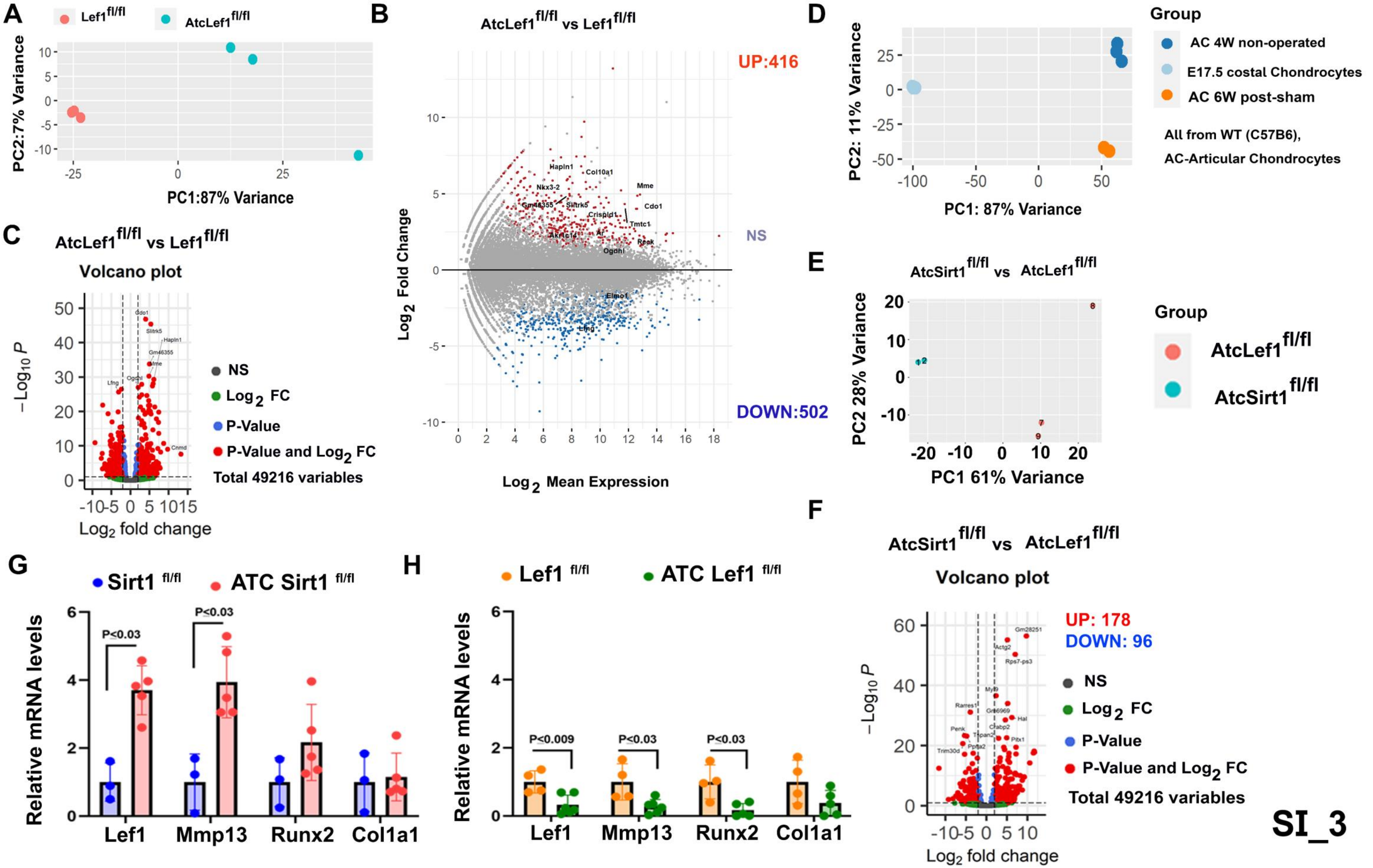

A
Lef1 fl/fl
AtcLef1 fl/fl
PC1:87% Variance
PC2:7% Variance
B
AtcLef1 fl/fl vs Lef1 fl/fl
UP:416
NS
DOWN:502
Log2 Fold Change
Log2 Mean Expression
Hapln1
Col10a1
Nkx3-2
Mme
Cdo1
Gm46355
Slitrk5
Crispld1
Tmtc1
Ar
Reck
Ogdhl
Elmo1
Lfng
C
AtcLef1 fl/fl vs Lef1 fl/fl
Volcano plot
−Log10 P
Log2 fold change
NS
Log2 FC
P-Value
P-Value and Log2 FC
Total 49216 variables
D
Group
AC 4W non-operated
E17.5 costal Chondrocytes
AC 6W post-sham
All from WT (C57B6),
AC-Articular Chondrocytes
PC1: 87% Variance
PC2: 11% Variance
E
AtcSirt1 fl/fl vs AtcLef1 fl/fl
Group
AtcLef1 fl/fl
AtcSirt1 fl/fl
PC1 61% Variance
PC2 28% Variance
F
AtcSirt1 fl/fl vs AtcLef1 fl/fl
Volcano plot
UP: 178
DOWN: 96
NS
Log2 FC
P-Value
P-Value and Log2 FC
Total 49216 variables
Log2 fold change
G
Sirt1 fl/fl
ATC Sirt1 fl/fl
Relative mRNA levels
P<0.03
P<0.03
Lef1
Mmp13
Runx2
Col1a1
H
Lef1 fl/fl
ATC Lef1 fl/fl
Relative mRNA levels
P≤0.009
P≤0.03
P≤0.03
Lef1
Mmp13
Runx2
Col1a1
SI_3

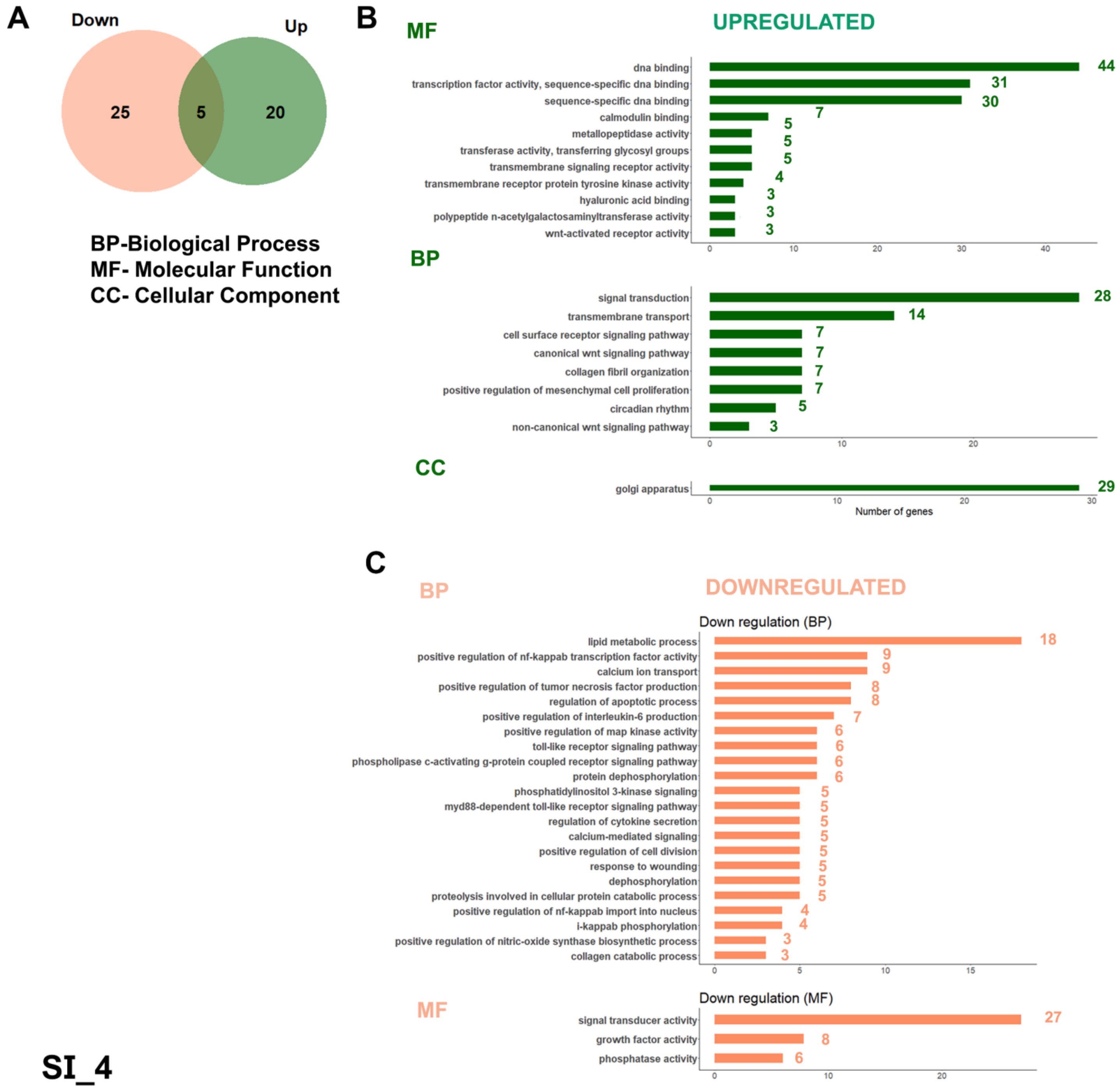
A
Down
Up
25
5
20
BP-Biological Process
MF- Molecular Function
CC- Cellular Component
B
MF
UPREGULATED
dna binding 44
transcription factor activity, sequence-specific dna binding 31
sequence-specific dna binding 30
calmodulin binding 7
metallopeptidase activity 5
transferase activity, transferring glycosyl groups 5
transmembrane signaling receptor activity 5
transmembrane receptor protein tyrosine kinase activity 4
hyaluronic acid binding 3
polypeptide n-acetylgalactosaminyltransferase activity 3
wnt-activated receptor activity 3
BP
signal transduction 28
transmembrane transport 14
cell surface receptor signaling pathway 7
canonical wnt signaling pathway 7
collagen fibril organization 7
positive regulation of mesenchymal cell proliferation 7
circadian rhythm 5
non-canonical wnt signaling pathway 3
CC
golgi apparatus 29
Number of genes
C
BP
DOWNREGULATED
Down regulation (BP)
lipid metabolic process 18
positive regulation of nf-kappab transcription factor activity 9
calcium ion transport 9
positive regulation of tumor necrosis factor production 8
regulation of apoptotic process 8
positive regulation of interleukin-6 production 7
positive regulation of map kinase activity 6
toll-like receptor signaling pathway 6
phospholipase c-activating g-protein coupled receptor signaling pathway 6
protein dephosphorylation 6
phosphatidylinositol 3-kinase signaling 5
myd88-dependent toll-like receptor signaling pathway 5
regulation of cytokine secretion 5
calcium-mediated signaling 5
positive regulation of cell division 5
response to wounding 5
dephosphorylation 5
proteolysis involved in cellular protein catabolic process 5
positive regulation of nf-kappab import into nucleus 4
i-kappab phosphorylation 4
positive regulation of nitric-oxide synthase biosynthetic process 3
collagen catabolic process 3
MF
Down regulation (MF)
signal transducer activity 27
growth factor activity 8
phosphatase activity 6

SI_4

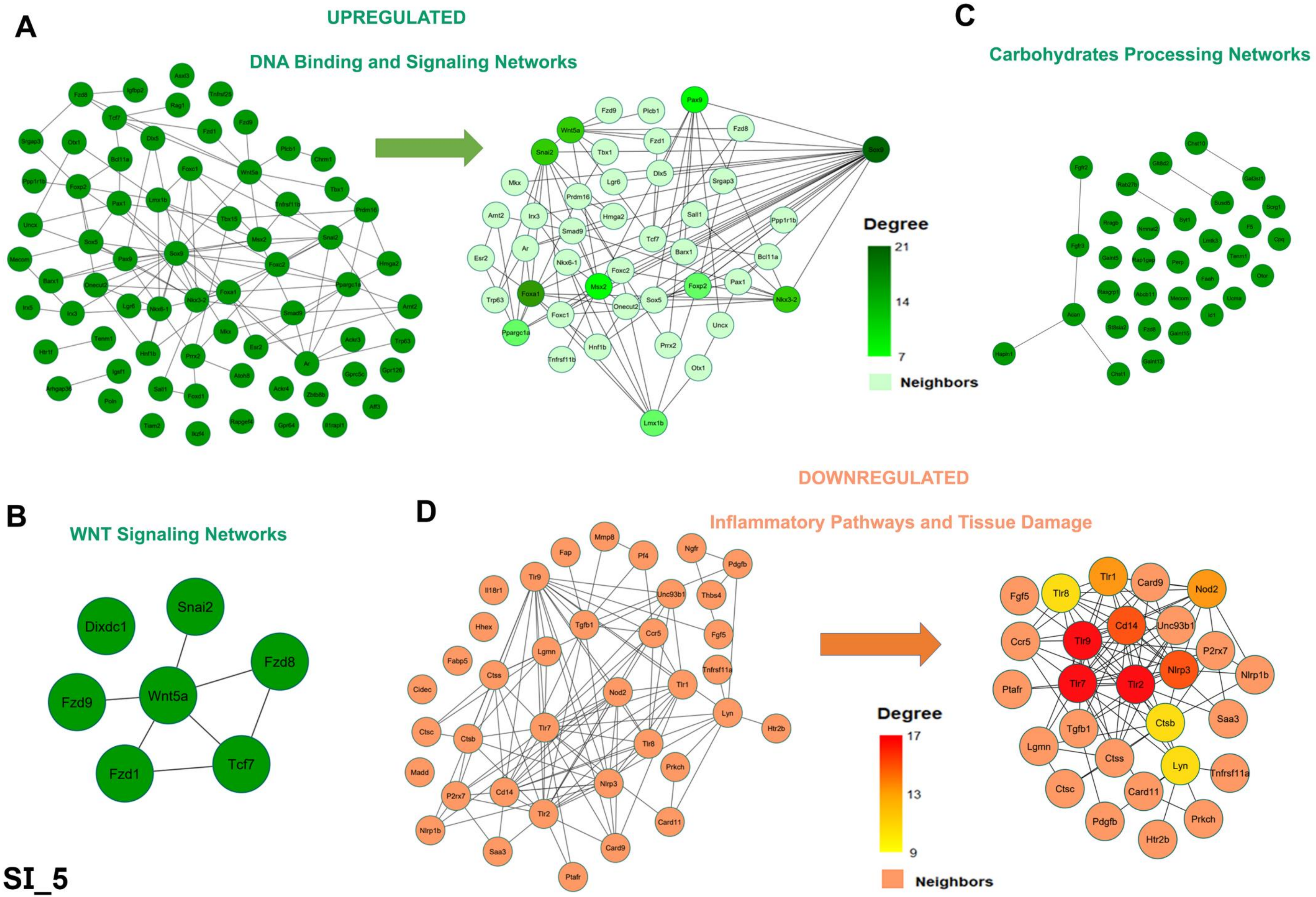

A
UPREGULATED
DNA Binding and Signaling Networks
Pax9
Fzd9
Plcb1
Wnt5a
Fzd8
Fzd1
Sox9
Snai2
Tbx1
Mkx
Lgr6
Dlx5
Srgap3
Prdm16
Hmga2
Arnt2
Irx3
Sall1
Ppp1r1b
Smad9
Tcf7
Ar
Barx1
Bcl11a
Esr2
Nkx6-1
Foxc2
Msx2
Foxp2
Pax1
Trp63
Foxa1
Onecut2
Sox5
Nkx3-2
Foxc1
Ppargc1a
Uncx
Hnf1b
Prrx2
Tnfrsf11b
Otx1
Lmx1b
Degree
21
14
7
Neighbors
C
Carbohydrates Processing Networks
B
WNT Signaling Networks
Snai2
Dixdc1
Fzd8
Wnt5a
Fzd9
Fzd1
Tcf7
DOWNREGULATED
D
Inflammatory Pathways and Tissue Damage
Mmp8
Fap
Pf4
Ngfr
Pdgfb
Tlr9
Il18r1
Unc93b1
Thbs4
Hhex
Tgfb1
Ccr5
Fgf5
Lgmn
Fabp5
Ctss
Tnfrsf11a
Cidec
Tlr1
Nod2
Lyn
Htr2b
Tlr7
Ctsc
Ctsb
Tlr8
Madd
Prkch
Nlrp3
P2rx7
Cd14
Card11
Tlr2
Nlrp1b
Card9
Saa3
Ptafr
Degree
17
13
9
Neighbors
Fgf5
Tlr8
Tlr1
Card9
Nod2
Cd14
Unc93b1
Ccr5
Tlr9
P2rx7
Nlrp3
Nlrp1b
Ptafr
Tlr7
Tlr2
Ctsb
Saa3
Tgfb1
Lgmn
Ctss
Lyn
Tnfrsf11a
Ctsc
Card11
Pdgfb
Prkch
Htr2b

SI_5

SI_6: Table a-d: Differentially altered genes by genes clusters for ATC Lef1$^{fl/fl}$ vs Lef1$^{fl/fl}$. Costal chondrocytes from ATC Lef1$^{fl/fl}$ vs Lef1$^{fl/fl}$ were isolated and proceed for RNAseq and subjected to bioinformatic analysis to establish differentially altered genes and related clusters via DAVID annotation (n=3, per genotype). Green highlight indicates upregulated gene, while pink highlight indices down regulated gene. ***SI_6*** is composed of 4 tables: **Table a**: Chondrogenic genes and Cartilage (DAVID); **Table b**: Fibrous collagens (DAVID); **Table c**: Catabolic (DAVID); **Table d**: Extracellular matrix pathways (DAVID). Green background for upregulated targets, orange for down regulated targets. Targets appear in ***Fig.3G*** in a z-score heatmap.

SI_6: Table a-d:

Differentially altered genes by genes clusters for ATC Lef1$^{fl/fl}$ vs Lef1$^{fl/fl}$.

Table a: Chondrogenic genes and Cartilage (DAVID)

Gene Symbol	Gene Name	Pvalue	Fold Change (Log2)
col9a1	collagen, type IX, alpha 1	1.82E-07	9.74*
otor	otoraplin	0.028987	6.88
acan	aggrecan	0.023858	6.38*
col10a1	collagen, type X, alpha 1	8.30E-25	5.76
col11a1	collagen, type XI, alpha 1	0.005569	2.32*
comp	cartilage oligomeric matrix protein	1.64E-14	3.51*
fgfr3	fibroblast growth factor receptor 3	0.021422	3.61*
sox5	SRY (sex determining region Y)-box 5	3.44E-05	3.29*
msx2	msh homeobox 2	0.000496	2.89
sox9	SRY (sex determining region Y)-box 9	0.056602	2.78*

Asterisks (*) denotes genes appearing under both "Chondrogenic" classification and "Cartilage" gene clusters based on DAVID annotation

Table b. Fibrous collagens

Gene Symbol	Gene Name	Pvalue	Fold Change (Log2)
col10a1	collagen, type X, alpha 1	8.30E-25	5.76
colec10	collectin sub-family member 10	0.000428	4.25
col23a1	collagen, type XXIII, alpha 1	0.01445	2.01
col8a1	collagen, type VIII, alpha 1	0.006815	1.82
colca2	COLCA2 homolog	0.002452	1.81
col15a1	collagen, type XV, alpha 1	3.67E-05	-3.47
col6a5	collagen, type VI, alpha 5	1.24E-08	-5.24

Table c. Catabolic

Gene Symbol	Gene Name	Pvalue	Fold Change (Log2)
mmp16	matrix metallopeptidase 16	5.34E-07	3.11
adamtsl3	ADAMTS-like 3	0.001408	2.81
adamtsl4	ADAMTS-like 4	0.000862	2.44
mmp8	matrix metallopeptidase 8	0.042197	-3.78

Table d. Extracellular matrix pathways (DAVID)

Gene Symbol	Gene Name	Pvalue	Fold Change (Log2)
hapln1	hyaluronan and proteoglycan link protein 1	1.76E-26	6.12
fgfr2	fibroblast growth factor receptor 2	2.41E-11	4.02
omd	osteomodulin	2.58E-10	4.11
mmp16	matrix metallopeptidase 16	5.34E-07	3.11
adamtsl4	ADAMTS-like 4	0.000862	2.44
ogn	osteoglycin	0.02959	2.41
lmcd1	LIM and cysteine-rich domains 1	0.099529	2.36
fn1	fibronectin 1	0.000236	2.25

ltbp1	latent transforming growth factor beta binding protein 1	0.011999	2.2
col8a1	collagen, type VIII, alpha 1	0.006815	1.82
lamc2	laminin, gamma 2	0.01247	-1.64
gldn	gliomedin	0.036717	-2.41
col15a1	collagen, type XV, alpha 1	3.67E-05	-3.47
mmp8	matrix metallopeptidase 8	0.042197	-3.78
spon1	spondin 1, (f-spondin) extracellular matrix protein	5.01E-09	-5.17
thbs4	thrombospondin 4	0.000882	-5.21
col6a5	collagen, type VI, alpha 5	1.24E-08	-5.24
cilp	cartilage intermediate layer protein, nucleotide pyrophosphohydrolase	1.32E-09	-5.31

SI_7: Table a-c: Differentially altered DAVID pathways for ATC Lef1$^{fl/fl}$ vs Lef1$^{fl/fl}$: Costal chondrocytes from ATC Lef1$^{fl/fl}$ vs Lef1$^{fl/fl}$ were isolated and proceed for RNAseq and subjected to bioinformatic analysis to establish differentially altered genes and related enriched clusters via DAVID annotation (n=3, per genotype). Green highlight indicates upregulated gene, while pink highlight indices down regulated gene. ***SI_7*** is composed of 3 tables**: Table a:** NFķB Signaling Pathways (DAVID); **Table b**: TGF/BMP and WNT Signaling Pathways (DAVID); **Table c**: Pain Pathways (DAVID). Green background for upregulated targets, orange for down regulated targets. Targets appear in ***Fig.5G*** in a z-score heatmap.

SI_7: Table a-d:

Differentially altered DAVID pathways for ATC Lef1$^{fl/fl}$ vs Lef1$^{fl/fl}$

Table a. NFκB Pathways (DAVID)

Gene Symbol	Gene Name	Pvalue	Fold Change (Log2)
nfkbid	nuclear factor of kappa light polypeptide gene enhancer in B cells inhibitor, delta	0.099817	-1.5
tgfb1	transforming growth factor, beta 1	0.051936	-1.54
tlr2	toll-like receptor 2	0.000288	-1.93
smpd3	sphingomyelin phosphodiesterase 3, neutral	0.002833	-2.65
arrb2	arrestin, beta 2	0.000241	-2.89
prkch	protein kinase C, eta	0.094915	-2.94
tnfrsf11a	tumor necrosis factor receptor superfamily, member 11a, NFKB activator	0.039558	-3
nod2	nucleotide-binding oligomerization domain containing 2	0.000203	-3.02
tlr7	toll-like receptor 7	0.075405	-3.13
nlrp3	NLR family, pyrin domain containing 3	0.086118	-3.24
rhoh	ras homolog family member H	0.041309	-3.31
card9	caspase recruitment domain family, member 9	0.021482	-3.32
card11	caspase recruitment domain family, member 11	0.058705	-3.58
il18r1	interleukin 18 receptor 1	0.034379	-3.64
tlr9	toll-like receptor 9	0.003027	-4.05
tnip3	TNFAIP3 interacting protein 3	0.062762	-4.17
adipoq	adiponectin, C1Q and collagen domain containing	0.004771	-5.62

Table b. TGF/BMP and WNT Signaling Pathways (DAVID)

Gene Symbol	Gene Name	Pvalue	Fold Change (Log2)
fzd9	frizzled class receptor 9	1.01E-11	7.45
smad9	SMAD family member 9	0.001363	2.91
wnt5a	wingless-type MMTV integration site family, member 5A	1.20E-08	2.34
fzd8	frizzled class receptor 8	0.049895	2.08
fzd1	frizzled class receptor 1	0.085898	1.66
tgfb2	transforming growth factor, beta 2	0.024561	1.82
tgfb1	transforming growth factor, beta 1	0.051936	-1.54
tgfbr1	transforming growth factor, beta receptor I	0.010356	-1.64

Table c. Pain Pathways (DAVID)

Gene Symbol	Gene Name	Pvalue	Fold Change (Log2)
cacna1a	calcium channel, voltage-dependent, P/Q type, alpha 1A subunit	0.03291	-1.63
p2rx7	purinergic receptor P2X, ligand-gated ion channel, 7	0.050407	-2.26
chrna4	cholinergic receptor, nicotinic, alpha polypeptide 4	0.062876	-3.2
lpar5	lysophosphatidic acid receptor 5	0.085566	-3.45
ednrb	endothelin receptor type B	0.004927	-3.59
osm	oncostatin M	0.019173	-3.78
grik1	glutamate receptor, ionotropic, kainate 1	0.000941	-4.06

ngfr	nerve growth factor receptor (TNFR superfamily, member 16)	4.27E-08	-5.03
thbs4	thrombospondin 4	0.000882	-5.21
tac1	tachykinin 1	0.004018	-6.78

SI_8: STRING analysis for GO Enrichment Networks for *ATC Lef1*$^{fl/fl}$ vs *Lef1*$^{fl/fl}$. Selected GO enrichment clusters were applied to STRING network analysis and displayed in 4 tables (**a-d**). STRING analysis was carried out according to the following parameters: conserved Neighborhood, co-occurrence, fusion co-expression, experiments, databases, text mining. **Table a**: Upregulated DNA binding and Signaling Networks (***SI_5A***), **Table b**: Upregulated WNT Signaling Networks (***SI_5B***); **Table c**: Upregulated Carbohydrate Processing Networks (***SI_5C***), **Table d**: Downregulated Inflammatory Pathways and Tissue Damage (***SI_5D***). The proteins appear in alphabetical order, except for the 10 most networked proteins, which appear in the top rows in dark background with their node degree, reflecting the extent of the networks. In the tables, dark green background represents the most networked upregulated genes, while dark orange represents the most networked downregulated genes, with the cluster. These targets are graphically illustrated in ***SI_5***.

SI_8: Table a-d
STRING analysis for GO Enrichment Networks for *ATC Lef1$^{fl/fl}$* vs *Lef1$^{fl/fl}$*.

Table a. Upregulated DNA binding and Signaling Networks (SI_5A)

Gene Symbol	Gene Name	Node Network ranking
**Sox9**	**SRY (sex determining region Y)-box 9**	**21**
**Foxa1**	**forkhead box A1**	**10**
**Wnt5a**	**wingless-type MMTV integration site family, member 5A**	**9**
**Snai2**	**snail family zinc finger 2**	**9**
**Nkx3-2**	**NK3 homeobox 2**	**9**
**Msx2**	**msh homeobox 2**	**8**
**Pax9**	**paired box 9**	**8**
**Lmx1b**	**LIM homeobox transcription factor 1 beta**	**7**
**Ppargc1a**	**peroxisome proliferative activated receptor, gamma, coactivator 1 alpha**	**7**
**Foxc2**	**forkhead box C2**	**7**
**Ackr3**	atypical chemokine receptor 3	
**Ackr4**	atypical chemokine receptor 4	
**Adgrg2**	adhesion G protein-coupled receptor G2	
**Adgrg6**	adhesion G protein-coupled receptor G6	
**Aff3**	AF4/FMR2 family, member 3	
**Ar**	androgen receptor	
**Arhgap36**	Rho GTPase activating protein 36	
**Arnt2**	aryl hydrocarbon receptor nuclear translocator 2	
**Asxl3**	ASXL transcriptional regulator 3	
**Atoh8**	atonal bHLH transcription factor 8	
**Barx1**	BarH-like homeobox 1	
**Bcl11a**	B cell CLL/lymphoma 11A (zinc finger protein)	
**Chrm1**	cholinergic receptor, muscarinic 1, CNS	
**Dlx5**	distal-less homeobox 5	
**Esr2**	estrogen receptor 2 (beta)	
**Foxc1**	forkhead box C1	
**Foxd1**	forkhead box D1	
**Foxp2**	forkhead box P2	
**Fzd1**	frizzled class receptor 1	
**Fzd8**	frizzled class receptor 8	
**Fzd9**	frizzled class receptor 9	
**Gprc5c**	G protein-coupled receptor, family C, group 5, member C	
**Hmga2**	high mobility group AT-hook 2	
**Hnf1b**	HNF1 homeobox B	
**Htr1f**	5-hydroxytryptamine (serotonin) receptor 1F	
**Igfbp2**	insulin-like growth factor binding protein 2	
**Igsf1**	immunoglobulin superfamily, member 1	
**Ikzf4**	IKAROS family zinc finger 4	
**Il1rapl1**	interleukin 1 receptor accessory protein-like 1	
**Irx3**	Iroquois related homeobox 3	
**Irx5**	Iroquois homeobox 5	
**Lgr6**	leucine-rich repeat-containing G protein-coupled receptor 6	
**Mecom**	MDS1 and EVI1 complex locus	
**Mkx**	mohawk homeobox	
**Nkx6-1**	NK6 homeobox 1	
**Onecut2**	one cut domain, family member 2	
**Otx1**	orthodenticle homeobox 1	

**Pax1**	paired box 1	
**Plcb1**	phospholipase C, beta 1	
**Poln**	DNA polymerase N	
**Ppp1r1b**	protein phosphatase 1, regulatory inhibitor subunit 1B	
**Prdm16**	PR domain containing 16	
**Prrx2**	paired related homeobox 2	
**Rag1**	recombination activating 1	
**Rapgef4**	Rap guanine nucleotide exchange factor (GEF) 4	
**Sall1**	spalt like transcription factor 1	
**Smad9**	SMAD family member 9	
**Sox5**	SRY (sex determining region Y)-box 5	
**Srgap3**	SLIT-ROBO Rho GTPase activating protein 3	
**Tbx1**	T-box 1	
**Tbx15**	T-box 15	
**Tcf7**	transcription factor 7, T cell specific	
**Tenm1**	teneurin transmembrane protein 1	
**Tiam2**	T cell lymphoma invasion and metastasis 2	
**Tnfrsf11b**	tumor necrosis factor receptor superfamily, member 11b (osteoprotegerin)	
**Tnfrsf25**	tumor necrosis factor receptor superfamily, member 25	
**Trp63**	transformation related protein 63	
**Uncx**	UNC homeobox	
**Zbtb8b**	zinc finger and BTB domain containing 8b	

Table b. Upregulated WNT Signaling Networks (SI_5B)

Gene Symbol	Gene Name
**Wnt5a**	**wingless-type MMTV integration site family, member 5A**
Dixdc1	DIX domain containing 1
Fzd8	frizzled class receptor 8
Fzd9	frizzled class receptor 9
Snai2	snail family zinc finger 2
Tcf7	transcription factor 7, T cell specific

Table c. Upregulated Carbohydrate Processing Networks (SI_5C)

Gene Symbol	Gene Name
**Acan**	**aggrecan**
Fgfr2	fibroblast growth factor receptor 2
Chst1	carbohydrate sulfotransferase 1
Chst10	carbohydrate sulfotransferase 10
Fgfr3	fibroblast growth factor receptor 3
Gal3st1	galactose-3-O-sulfotransferase 1
Glt8d2	glycosyltransferase 8 domain containing 2
Hapln1	hyaluronan and proteoglycan link protein 1
Rab27b	RAB27B, member RAS oncogene family
Susd5	sushi domain containing 5
Syt1	synaptotagmin I
Abcb11	ATP-binding cassette, sub-family B (MDR/TAP), member 11
Cpq	carboxypeptidase Q
F5	coagulation factor V
Faah	fatty acid amide hydrolase
Fzd8	frizzled class receptor 8
Galnt13	polypeptide N-acetylgalactosaminyltransferase 13
Galnt15	polypeptide N-acetylgalactosaminyltransferase 15
Id1	inhibitor of DNA binding 1, HLH protein

Lmtk3	lemur tyrosine kinase 3
Mecom	MDS1 and EVI1 complex locus
Nmnat2	nicotinamide nucleotide adenylyltransferase 2
Otor	otoraplin
Perp	PERP, TP53 apoptosis effector
Rap1gap	Rap1 GTPase-activating protein
Rasgrp1	RAS guanyl releasing protein 1
Rragb	Ras-related GTP binding B
Scrg1	scrapie responsive gene 1
St8sia2	ST8 alpha-N-acetyl-neuraminide alpha-2,8-sialyltransferase 2
Tenm1	teneurin transmembrane protein 1
Ucma	upper zone of growth plate and cartilage matrix associated

Table d. Downregulated Inflammatory Pathways and Tissue Damage (SI_5D)

Gene Symbol	Gene Name	Node Network ranking
**Tlr2**	**toll-like receptor 2**	**17**
**Tlr7**	**toll-like receptor 7**	**17**
**Tlr9**	**toll-like receptor 9**	**17**
**Nlrp3**	**NLR family, pyrin domain containing 3**	**12**
**Cd14**	**CD14 antigen**	**12**
**Nod2**	**nucleotide-binding oligomerization domain containing 2**	**10**
**Tlr1**	**toll-like receptor 1**	**10**
**Tlr8**	**toll-like receptor 8**	**9**
**Lyn**	**LYN proto-oncogene, Src family tyrosine kinase**	**9**
**Ctsb**	**cathepsin B**	**9**
**Card11**	caspase recruitment domain family, member 11	
**Card9**	caspase recruitment domain family, member 9	
**Ccr5**	chemokine (C-C motif) receptor 5	
**Cidec**	cell death-inducing DFFA-like effector c	
**Ctsc**	cathepsin C	
**Ctss**	cathepsin S	
**Fabp5**	fatty acid binding protein 5, epidermal	
**Fgf5**	fibroblast growth factor 5	
**Hhex**	hematopoietically expressed homeobox	
**Htr2b**	5-hydroxytryptamine (serotonin) receptor 2B	
**Il18r1**	interleukin 18 receptor 1	
**Lgmn**	legumain	
**Madd**	MAP-kinase activating death domain	
**Mmp8**	matrix metallopeptidase 8	
**Ngfr**	nerve growth factor receptor (TNFR superfamily, member 16)	
**Nlrp1b**	NLR family, pyrin domain containing 1B	
**Nod2**	nucleotide-binding oligomerization domain containing 2	
**P2rx7**	purinergic receptor P2X, ligand-gated ion channel, 7	
**Pdgfb**	platelet derived growth factor, B polypeptide	
**Pf4**	platelet factor 4	
**Prkch**	protein kinase C, eta	
**Ptafr**	platelet-activating factor receptor	
**Saa3**	serum amyloid A 3	
**Tgfb1**	transforming growth factor, beta 1	
**Thbs4**	thrombospondin 4	
**Tnfrsf11a**	tumor necrosis factor receptor superfamily, member 11a, NFKB activator	
**Unc93b1**	unc-93 homolog B1, TLR signaling regulator	

SI_9:PNAS_2021116855_s2.xlsx

Excel Dataset; Elayyan et al

SI 10: Extracellular Region GO Enrichment cluster for *ATC Sirt11$^{fl/fl}$* vs *ATC Lef1$^{fl/fl}$*:

Costal chondrocytes from ATC Sirt11$^{fl/fl}$ (n=2) vs ATC Lef1$^{fl/fl}$ (n=3) transgenes were isolated and processed for RNAseq and subjected to bioinformatic as indicated in the materials and methods section. GO enrichment gene cluster was obtained for the Extracellular Region classification, to determine differentially expressed genes that may be secreted from cartilage**. Table a**. Upregulated genes with the 10 most differentially increased LFC in dark green highlight, **Table b.** Downregulated genes with the 10 most differentially decreased LFC in dark orange highlight (***Fig. 4G*** graphical depiction).

SI_10: Table a,b

Extracellular Region GO Enrichment cluster for *ATC Sirt11$^{fl/fl}$* vs *ATC Lef1$^{fl/fl}$*

Table a. Upregulated Genes

Gene Symbol	Gene Name	Log Fold Change
**Gbp2b**	**guanylate binding protein 2b**	**11.73898**
**Fgf5**	**fibroblast growth factor 5**	**8.748668**
**Serpina3k**	**serine (or cysteine) peptidase inhibitor, clade A, member 3K**	**7.701685**
**Serpina3m**	**serine (or cysteine) peptidase inhibitor, clade A, member 3M**	**7.032729**
**Cilp**	**cartilage intermediate layer protein, nucleotide pyrophosphohydrolase**	**6.352912**
**Cxcl9**	**chemokine (C-X-C motif) ligand 9**	**6.189629**
Mmp8	matrix metallopeptidase 8	5.132841
Thbs4	thrombospondin 4	4.473944
Pnp2	purine-nucleoside phosphorylase 2	4.187719
Pm20d1	peptidase M20 domain containing 1	3.961473
**Cck**	cholecystokinin	3.851476
**Apol10b**	apolipoprotein L 10B	3.802427
**Gldn**	gliomedin	3.752421
**Crispld2**	cysteine-rich secretory protein LCCL domain containing 2	3.559773
**Serpine1**	serine (or cysteine) peptidase inhibitor, clade E, member 1	3.166668
**Serpina3n**	serine (or cysteine) peptidase inhibitor, clade A, member 3N	3.000549
**Pla1a**	phospholipase A1 member A	2.816846
**Thsd7a**	thrombospondin, type I, domain containing 7A	2.721571
**Enho**	energy homeostasis associated	2.617786
**Angptl7**	angiopoietin-like 7	2.565991
**Tnc**	tenascin C	2.390366
**Angpt4**	angiopoietin 4	2.331636
**Fstl3**	follistatin-like 3	2.031144

Table b. Downregulated Genes

Gene Symbol	Gene Name	Log Fold Change
**Cd5l**	**CD5 antigen-like**	**-6.96642**
Col10a1	collagen, type X, alpha 1	-5.2931
Penk	preproenkephalin	-5.27742
C4bp	complement component 4 binding protein	-5.11793
Cfi	complement component factor i	-5.05504
Il17rb	interleukin 17 receptor B	-4.69759
Matn4	matrilin 4	-3.9508
Igfals	insulin-like growth factor binding protein, acid labile subunit	-3.91398
Cxcl15	chemokine (C-X-C motif) ligand 15	-3.69296
Tenm1	teneurin transmembrane protein 1	-3.448
**Fgfr2**	fibroblast growth factor receptor 2	-3.32097
**Lama3**	laminin, alpha 3	-3.26376
**Itih2**	inter-alpha trypsin inhibitor, heavy chain 2	-2.68547
**Ogn**	osteoglycin	-2.59899
**Cxcl12**	chemokine (C-X-C motif) ligand 12	-2.56461

**Fam180a**	family with sequence similarity 180, member A	-2.37576
**Bmp4**	bone morphogenetic protein 4	-2.17422
**Prss35**	protease, serine 35	-2.04908
**Col23a1**	collagen, type XXIII, alpha 1	-2.01663
**Sema3f**	sema domain, immunoglobulin domain (Ig), short basic domain, secreted, (semaphorin) 3F	-1.96853

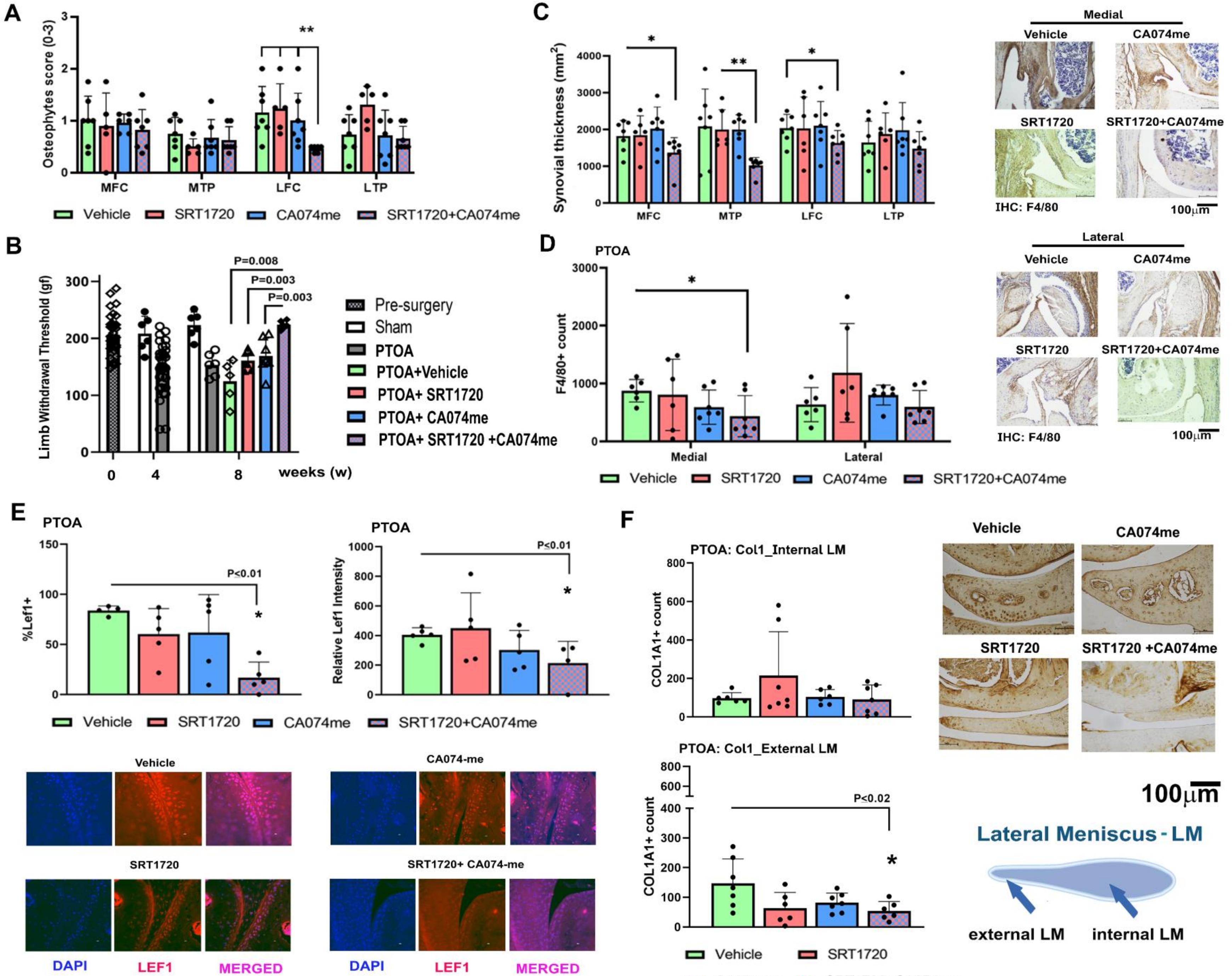
A
Osteophytes score (0-3)
MFC
MTP
LFC
LTP
**
Vehicle
SRT1720
CA074me
SRT1720+CA074me
B
Limb Withdrawal Threshold (gf)
P=0.008
P=0.003
P=0.003
weeks (w)
Pre-surgery
Sham
PTOA
PTOA+Vehicle
PTOA+ SRT1720
PTOA+ CA074me
PTOA+ SRT1720 +CA074me
C
Synovial thickness (mm²)
Medial
Vehicle
CA074me
SRT1720
SRT1720+CA074me
IHC: F4/80
100μm
D
PTOA
F4/80+ count
Medial
Lateral
E
PTOA
%Lef1+
P<0.01
Relative Lef1 Intensity
P≤0.01
CA074-me
SRT1720+ CA074-me
DAPI
LEF1
MERGED
F
PTOA: Col1_Internal LM
COL1A1+ count
PTOA: Col1_External LM
P<0.02
SRT1720 +CA074me
Lateral Meniscus - LM
external LM
internal LM

SI_11

SI_12

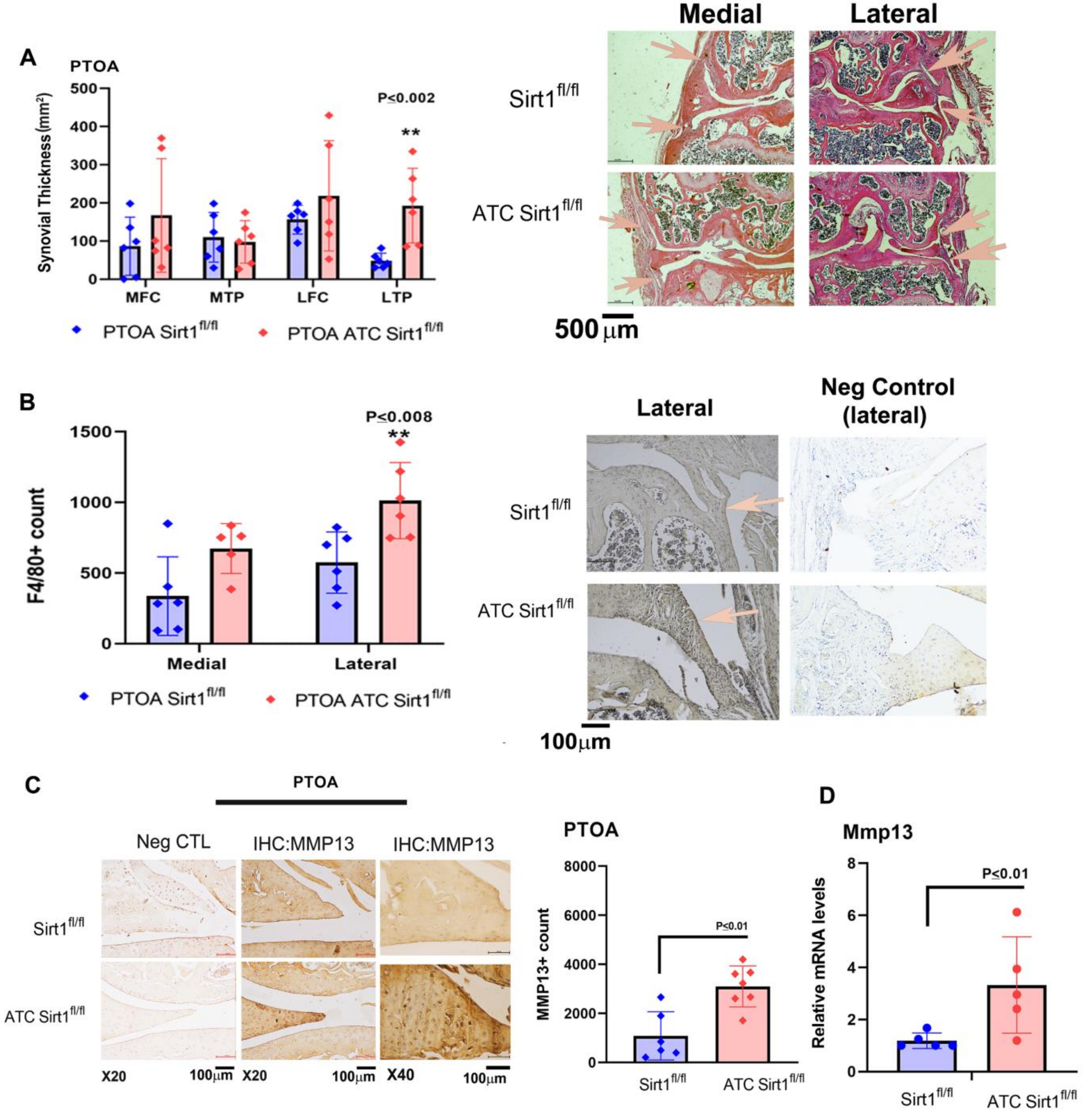

A
PTOA
Synovial Thickness (mm²)
P≤0.002
**
MFC
MTP
LFC
LTP
PTOA Sirt1 fl/fl
PTOA ATC Sirt1 fl/fl
PTOA
Medial
Lateral
Sirt1 fl/fl
ATC Sirt1 fl/fl
500 µm
B
F4/80+ count
P≤0.008
**
Medial
Lateral
PTOA Sirt1 fl/fl
PTOA ATC Sirt1 fl/fl
Lateral
Neg Control (lateral)
Sirt1 fl/fl
ATC Sirt1 fl/fl
100µm
C
PTOA
Neg CTL
IHC:MMP13
IHC:MMP13
Sirt1 fl/fl
ATC Sirt1 fl/fl
X20
100µm
X20
100µm
X40
100µm
PTOA
MMP13+ count
P≤0.01
Sirt1 fl/fl
ATC Sirt1 fl/fl
D
Mmp13
Relative mRNA levels
P≤0.01
Sirt1 fl/fl
ATC Sirt1 fl/fl

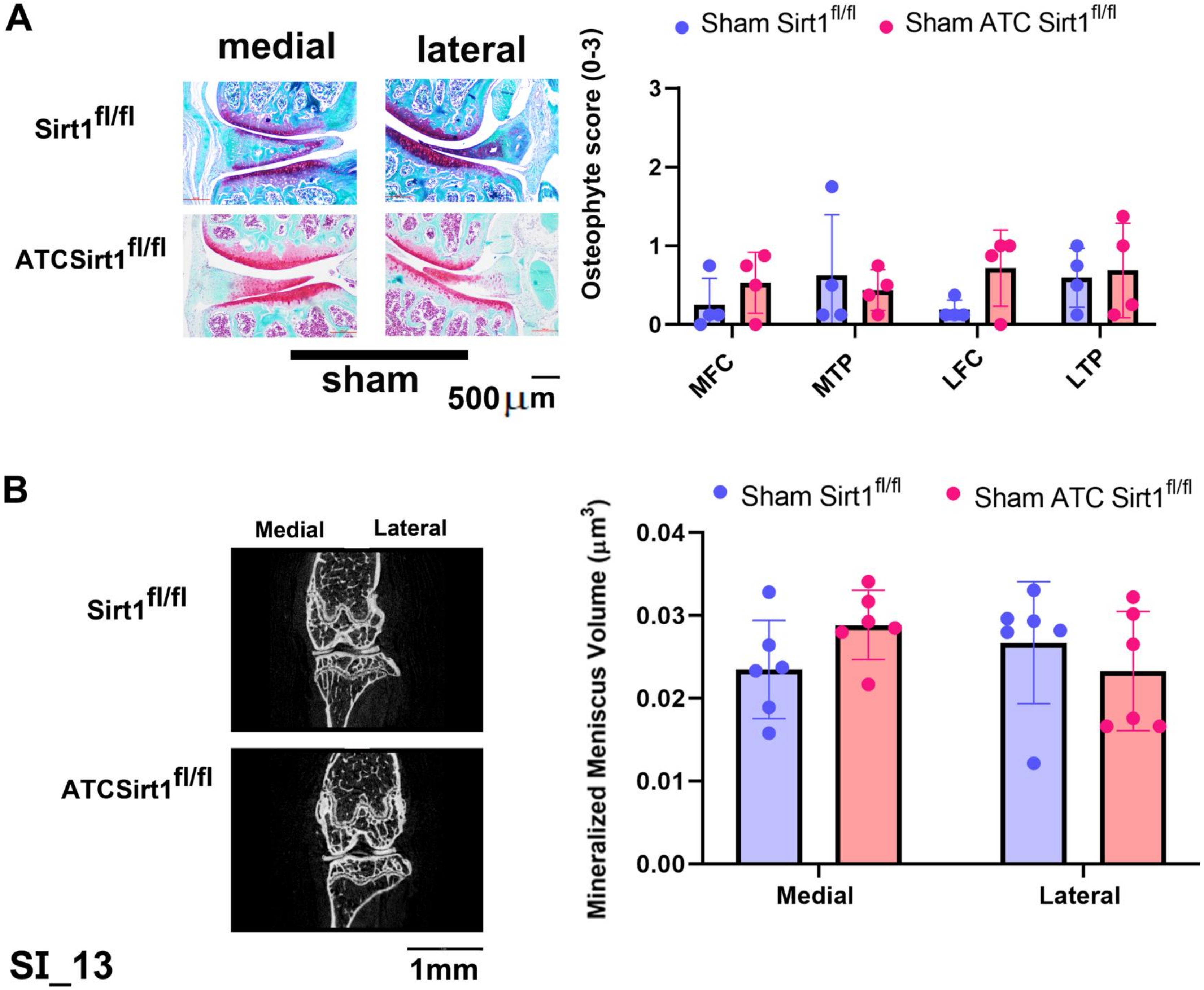
A
medial
lateral
Sirt1fl/fl
ATCSirt1fl/fl
sham
500 μm
Sham Sirt1fl/fl
Sham ATC Sirt1fl/fl
Osteophyte score (0-3)
3
2
1
0
MFC
MTP
LFC
LTP
B
Medial
Lateral
Sirt1fl/fl
ATCSirt1fl/fl
1mm
Sham Sirt1fl/fl
Sham ATC Sirt1fl/fl
Mineralized Meniscus Volume (μm3)
0.04
0.03
0.02
0.01
0.00
Medial
Lateral

SI_13

SI_14

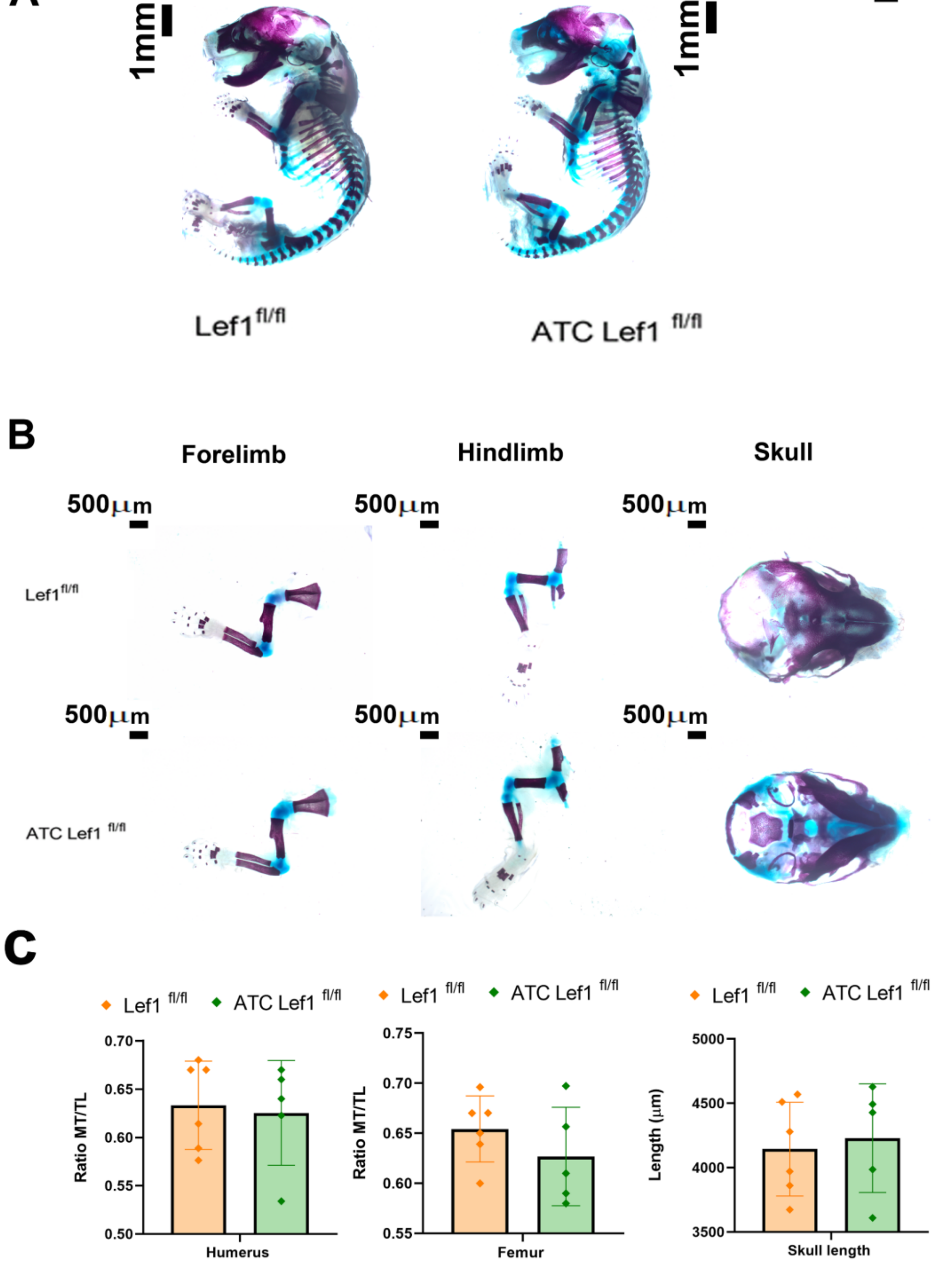

A
1mm
1mm
Lef1 fl/fl
ATC Lef1 fl/fl
B
Forelimb
Hindlimb
Skull
500µm
500µm
500µm
Lef1 fl/fl
500µm
500µm
500µm
ATC Lef1 fl/fl
C
Lef1 fl/fl
ATC Lef1 fl/fl
Ratio MT/TL
0.70
0.65
0.60
0.55
0.50
Humerus
Lef1 fl/fl
ATC Lef1 fl/fl
Ratio MT/TL
0.75
0.70
0.65
0.60
0.55
Femur
Lef1 fl/fl
ATC Lef1 fl/fl
Length (µm)
5000
4500
4000
3500
Skull length

SI_15

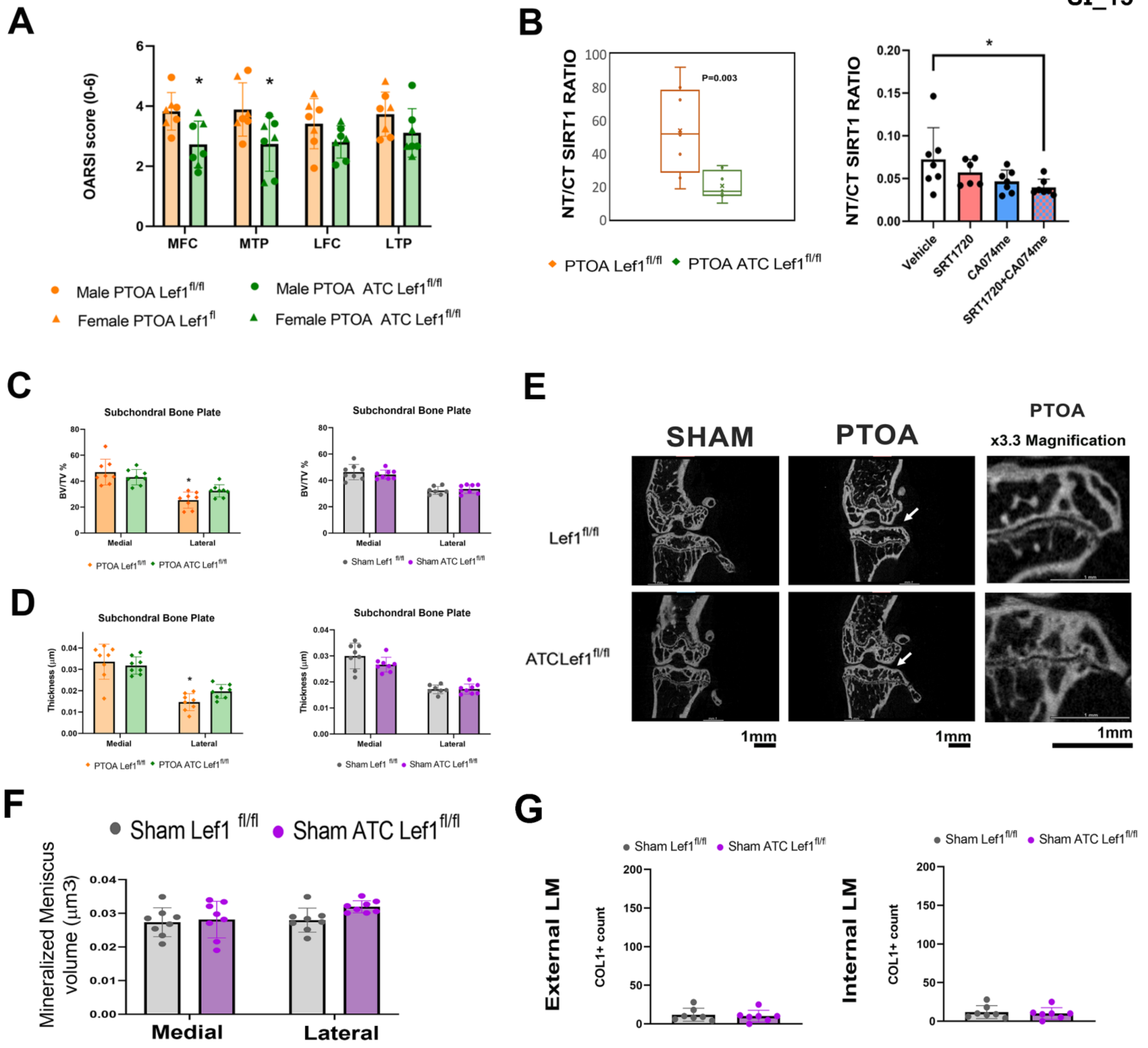

A
OARSI score (0-6)
MFC
MTP
LFC
LTP
Male PTOA Lef1 fl/fl
Female PTOA Lef1 fl
Male PTOA ATC Lef1 fl/fl
Female PTOA ATC Lef1 fl/fl
B
NT/CT SIRT1 RATIO
P=0.003
PTOA Lef1 fl/fl
PTOA ATC Lef1 fl/fl
Vehicle
SRT1720
CA074me
SRT1720+CA074me
C
Subchondral Bone Plate
BV/TV %
Medial
Lateral
PTOA Lef1 fl/fl
PTOA ATC Lef1 fl/fl
Sham Lef1 fl/fl
Sham ATC Lef1 fl/fl
D
Subchondral Bone Plate
Thickness (µm)
Medial
Lateral
E
SHAM
PTOA
PTOA x3.3 Magnification
Lef1 fl/fl
ATCLef1 fl/fl
1mm
F
Sham Lef1 fl/fl
Sham ATC Lef1 fl/fl
Mineralized Meniscus volume (µm3)
Medial
Lateral
G
External LM
Internal LM
COL1+ count
Sham Lef1 fl/fl
Sham ATC Lef1 fl/fl

Figure Legends of Supplemental information (SI)

SI_1: Materials and Methods

SI_2: Primer tables and sex related OA severity of transgenes: Composed of four tables (**a-d**): **Table a**: Primers of Lef1 Gene; **Table b**: OA severity between males and females of Sirt1 transgenes; OA severity between males and females of the same genotype and surgical procedure, based on Glasson et al., 2010 grading, and Mann Whitney U test (p>0.05). **Table c**: OA severity between males and females of Lef1 transgenes. OA severity between males and females of the same genotype and surgical procedure, based on Glasson et al., 2010 grading, and Mann Whitney U test (p>0.05); **Table d**: PCR and genotyping primers.

SI_3: Bioinformatic profiling of bulk RNA sequencing data. mRNA was extracted from ribcage E17 chondrocytes bearing a *Lef1* $^{fl/fl}$ and *ATC Lef1* $^{fl/fl}$ genotype (n=3), and processed through a pipeline detailed in the Materials and Methods clause. (**A**) Principle component analysis (PCA) of *Lef1* $^{fl/fl}$ and *ATC Lef1* $^{fl/fl}$ exhibits that the three biological replicates belonging to *Lef1* $^{fl/fl}$ (red) separated from the three biological replicates of *ATC Lef1* $^{fl/fl}$ (blue). **(B, C**) exhibit log fold change of genes in MA plot, or volcano plot (49216 total gene variables), respectively. Volcano plot displays statistical significance (P-value) versus magnitude of change (fold change) for differentially expressed genes. (**D**) denotes PCA comparisons between rib chondrocytes obtained from Lef1$^{fl/fl}$ (n=3) with samples from same C57BL6 background derived from deposited GEO databanks for adult articular chondrocytes derived from 3-6 month C57BL/6 mice, either post-sham procedure (GSE143447, n=2) or obtained from un-operated joint (GSE175486 **,**n=3). The comparison between costal chondrocytes and articular chondrocytes established a minor variance in PC2, yet a more pronounced variation on the PC1, which is the reason for our assessment of expression profiles amongst both cell

types, within the results section. (**E**) Displays a PCA plot of *ATC Lef1* $^{fl/fl}$ (n=3, red) and *ATC Sirt1* $^{fl/fl}$ (n=2, blue), **(F)** Displays a volcano plot (49216 total gene variables), for ATC Lef1 $^{fl/fl}$ (n=3) and ATC Sirt1 $^{fl/fl}$ (n=2). Volcano plot displays statistical significance (P-value) versus magnitude of change (fold change) for differentially expressed genes. (**G-H**) Articular chondrocytes from flox and cartilage-specific null transgenes were obtained at 3 months of age and analyzed for quantitative PCR of *Lef1*, *Mmp13* and *Runx2* and Col1a1 for each transgene; (**G**) *ATC Sirt1* $^{fl/fl}$ (n=5) vs *Sirt1* $^{fl/fl}$ controls (n=3); or (**H**) *ATC Lef1* $^{fl/fl}$ (n=6) vs *Lef1* $^{fl/fl}$ controls (n=4).

SI_4: Gene ontology (GO) enrichment clusters of *Lef1* $^{fl/fl}$ and *ATC Lef1* $^{fl/fl}$. Gene ontology enrichment clusters were obtained and subjected to a Venn diagram to determine uniquely upregulated and downregulated pathways (**A**). Next, unique GO enrichment clusters for upregulated (**B**) and downregulated (**C**) of the ATC *Lef1* $^{fl/fl}$ vs *Lef1* $^{fl/fl}$ controls, were segregated to categories- BP (Biological processes); MF (Molecular function) and CC (Cellular component).

SI_5: STRING analysis for GO enriched clusters of *Lef1* $^{fl/fl}$ and *ATC Lef1* $^{fl/fl}$. Upregulated clusters were divided into 3 groups and plotted in a STRING diagrams (green spheres). DNA binding and Signaling (**A**) with further analysis of the 10 most networked proteins along with their neighbors (arrow in **A**), WNT pathways (**B**) and carbohydrate processing (**C**). Similarly, downregulated STRING networks (orange spheres) are placed on a single STRING diagram (**D**) and further analyzed for the 10 most networked proteins along with their neighbors (arrow in **D**). Individual pathways present color-coded intensity for most networked proteins (amongst the 10 networked proteins) based on protein degree, referenced as "*Inflammatory*

*Pathways and Tissue Damag*e". Genes are listed in tables comprising the 10 most networked protein and entire gene network within the combined GO enrichment clusters are (**SI_8**).

SI_6: Differentially altered genes by genes clusters for *ATC Lef1$^{fl/fl}$* vs *Lef1$^{fl/fl}$*. Costal chondrocytes from ATC Lef1$^{fl/fl}$ vs Lef1$^{fl/fl}$ were isolated and proceed for RNAseq and subjected to bioinformatic analysis to establish differentially altered genes and related clusters via DAVID annotation (n=3, per genotype). Green highlight indicates upregulated gene, while pink highlight indices down regulated gene. ***SI_6*** is composed of 4 tables: **Table a**: Chondrogenic genes and Cartilage (DAVID); **Table b**: Fibrous collagens (DAVID); **Table c**: Catabolic (DAVID); **Table d**: Extracellular matrix pathways (DAVID). Green background for upregulated targets, orange for down regulated targets. Targets appear in ***Fig.3G*** in a z-score heatmap.

SI_7: Enriched DAVID pathways for *ATC Lef1$^{fl/fl}$* vs *Lef1$^{fl/fl}$*: Costal chondrocytes from *ATC Lef1$^{fl/fl}$* vs *Lef1$^{fl/fl}$* were isolated and processed for RNAseq and subjected to bioinformatic analysis to establish differentially altered genes and related enriched clusters via DAVID annotation (n=3, per genotype). Green highlight indicates upregulated gene, while pink highlight indices down regulated gene. ***SI_7*** is composed of 3 tables: ***Table a****. NFķB Signaling Pathways (DAVID);* ***Table b****. TGF/BMP and WNT Signaling Pathways (DAVID);* ***Table c****. Pain Pathways (DAVID).* Green background for upregulated targets, orange for down regulated targets. Targets appear in **Fig. 5G** in a z-score heatmap.

SI_8: STRING analysis for GO Enrichment Networks for *ATC Lef1$^{fl/fl}$* vs *Lef1$^{fl/fl}$*. Selected GO enrichment clusters were applied to STRING network analysis and displayed in 4 tables

(**a-d**). STRING analysis was carried out according to the following parameters: conserved Neighborhood, co-occurrence, fusion co-expression, experiments, databases, text mining. **Table a:** Upregulated DNA binding and Signaling Networks (***SI_5A***), **Table b**: Upregulated WNT Signaling Networks (***SI_5B***); **Table c**: Upregulated Carbohydrate Processing Networks (***SI_5C***), **Table d**: Downregulated Inflammatory Pathways and Tissue Damage (***SI_5D***). The proteins appear in alphabetical order, except for the 10 most networked proteins, which appear in the top rows in dark background with their node degree, reflecting the extend of the networks. In the tables, dark green background represents the most networked upregulated genes, while dark orange represents the most networked downregulated genes, with the cluster. These targets are graphically illustrated in ***SI_5***.

SI_9: Upregulated and downregulated genes for *ATC Lef1$^{fl/fl}$* vs *Lef1$^{fl/fl}$*. Differentially expressed genes exhibiting a Log fold change of >1 are displayed in an excel file, with five separated section; First section to the left is ***SI_9*** Legend, next section lists "Raw Up genes ATC-LEF1" with a total of 415 upregulated genes, some highlighted in red font and appear under the adjacent sheet labeled" Upregulated-Referenced" (59 genes). Under this excel sheet, upregulated genes that are associated with Osteoarthritis, Calcified tissue or Cartilage (based on PubMed search) are referenced using PMID annotation and a short description. Grey highlighted genes (28) are correlated with OA and appear upregulated in Lef1 nulls. The remainder upregulated genes (31 referenced genes) are associated with protective OA-related effects and partially illustrated in Fig. 6E scheme. Similarly, in the fourth sheet to left, we display a total of 491 downregulated genes with a Log fold change of <-1 are listed under "Raw Down genes ATC-LEF1", some highlighted in red font and appear under "Downregulated-Referenced" (44 genes). Under this excel sheet, downregulated genes that are associated with Osteoarthritis or cartilage (based on PubMed search) are referenced

using PMID annotation and a short description. Grey genes (8) are protective of OA and appear downregulated in Lef1 nulls, while the remainder downregulated genes (36 referenced genes) have been associated with OA damage, and partially illustrated in Fig. 6E.

SI_10: Extracellular Region GO Enrichment clusters for *ATC Sirt11$^{fl/fl}$* vs *ATC Lef1$^{fl/fl}$*: Costal chondrocytes from *ATC Sirt11$^{fl/fl}$* (n=2) vs *ATC Lef1$^{fl/fl}$* (n=3) transgenes were isolated and processed for RNAseq and subjected to bioinformatic as indicated in the materials and methods section. GO enrichment gene clusters were obtained for the *Extracellular Region* classification, to determined differentially expressed genes that may be secreted from cartilage. **Table a**. Upregulated genes with the 10 most differentially increased LFC in dark green highlight, **Table b.** Downregulated genes with the 10 most differentially decreased LFC in dark orange highlight (***Fig. 4G*** graphical depiction).

SI_11: Combined activation of Sirt1 and blockage of cathepsin B prevented PTOA: Here we employed an experimental model protrayed in **Fig. 1F**. Breifly, PTOA mice (Female, three months old, n≥5-7 per group) were injected twice a week with vehicle (20 %v/v DMSO in PBS), SRT1720 and\or CA074me, from week 4-8 post surgical procedure, as indicated in Materials and Methods. (**A**) Osteophyte score of all compartments (0-3); 8 weeks PTOA: SRT1720 and CA-074me (purple bar), SRT1720 (red bar), CA-074me (blue bar) and vehicle (green bar); (**B**) PAM measurments for joint pain are conducted at 3 points during the experiment: PTOA - 0 weeks, before the first injection - 4 weeks and 8 weeks after PTOA, prior to euthanizing the mice. Sham (white bars); PTOA (grey bars). **(C)** Synovial thickness and **(D) F4/80** positive cells in synovial memebrane; (**E**) Lef1 immunostaining; Images were quantified for percent of cells positive for DAPI and LEF1 co-appearance (left graph); or LEF1

intensity within the DAPI positive nuclei (right graph) of PTOA mice treated with vehicle, SRT1720, CA074me, and SRT1720 + CA074me. Representative immunofluorescent images are presented to the right of the graphs (x40 magnification). (**F**) Collgen type 1 staining for the lateral menisci of PTOA mice treated with vehicle, SRT1720, CA074Me, and SRT1720 + CA074me. Left graph is of the internal region and right graph is of the external region (illustration on the right of the representative immune-stained images). Statistical significance determined by Kruskal Wallis test followed by Mann and Whitney for paired treatments, assuming confidence for $p<0.05$ (*) and $p<0.001$ (**).

SI_12: PTOA induced *ATC Sirt1$^{fl/fl}$* mice display increased Mmp13 and augmented lateral synovitis. (**A**) Graph depicting synovial thickness (mm^2), for all joint compartments (n=6). Right panel are representative H&E sections of synovial lining from PTOA *Sirt1$^{fl/fl}$* and *ATC Sirt1$^{fl/fl}$* mice (pink arrows). (**B**) F4/80 positive cells in the synovial lining of PTOA *Sirt1$^{fl/fl}$* and *ATC Sirt1$^{fl/fl}$* mice. Right panel images exhibit the F4/80 staining of the synovial lining for the medial and lateral compartments. (**C**) IHC sections of lateral menisci from PTOA *Sirt1$^{fl/fl}$* and *ATC Sirt1$^{fl/fl}$* mice, stained for MMP13 vs. a negative control (left panels; Neg CTL). Graph to the right quantifies the intensity of MMP13 staining via ImageJ analysis for the lateral menisci. (**D**) Costal chondrocytes were isolated from E17 *Sirt1$^{fl/fl}$* and *ATC Sirt1$^{fl/fl}$* mice (n=5) and analyzed for *Mmp13* expression.

SI_13: Sham *Sirt1$^{fl/fl}$* and *ATC Sirt1$^{fl/fl}$* mice do not exhibit osseous remodeling in both compartments. (**A**) Osteophyte formation was quantified in post-sham *Sirt1$^{fl/fl}$* and *ATC Sirt1$^{fl/fl}$* mice PTOA (*Sirt1$^{fl/fl}$* n=6 female n=3; male n=3, *ATC Sirt1$^{fl/fl}$* n=9 female n=4; male n=5) Left panel exhibits representative Safranin-O/Fast green stained joint sections of medial

tibial plateau (MTP) or lateral tibial plateau (LTP) or medial femoral condyle (MFC) or lateral femoral condyle (LFC). (**B**) 3D μCT joint reconstructions of Sham mice (left panel) show calcified menisci colored white of *Sirt1$^{fl/fl}$* and *ATC Sirt1$^{fl/fl}$* mice and quantified (right panel) for the volume of calcified meniscus (μm^3) between experimental groups (n=6 each, female n=3; male n=3). All experiments were subjected to Kruskal-Wallis and post hoc Dunn's test to assess differences between multiple experimental groups, assuming a confidence level of 95% ($p < 0.05$). To examine statistical significance between mice genotypes post-sham, we employed Mann-Whitney U test, considering $p < 0.05$ (denoted *) to be statistically significant.

SI_14: Cartilage specific Lef1 ablated embryos do not exhibit a growth phenotype. (**A**) Whole skeletal segments from E17 littermate embryos were obtained from pregnant dams induced with Doxycycline (1 week prior to sacrifice); Right panel *Lef1$^{fl/fl}$* and Left panel *ATC Lef1$^{fl/fl}$* stained via alizarin red and Alcian blue (*Lef1$^{fl/fl}$* n=5, *ATC Sirt1$^{fl/fl}$* n=6), whole skeletal depictions. (**B**) Left panel shows forelimb, middle panel shows hindlimb and right panel shows the skull. (**C**) depicts graphs that have the Mineralized Tissue (MT; red stain) vs Total Length (TL; blue and red stained segment) quantified for humeri, femur and skull.

SI_15: **Joint and Bone Characteristics of in PTOA *Lef1$^{fl/fl}$* vs *ATC Lef1$^{fl/fl}$*.** (**A**) OA severity ranking between PTOA *Lef1$^{fl/fl}$* (n=7; female n=3; male n=4) and *ATC Lef1$^{fl/fl}$* (n=7; female n=4; male n=3), from 3 spaced (80μm space) tibiofemoral sections, which were graded for OA severity (0-6, Glasson et al., 2010). The additional sections cumulatively maintained a statistically significant trend, indicating reduced medial severity for the ATC Lef1$^{fl/fl}$ mice PTOA**. (B)** Serum NT/CT SIRT1 Ratio measured for PTOA *Lef1$^{fl/fl}$* and *ATC Lef1$^{fl/fl}$* (left graph) and combination treatment mice (e.g. Fig 1F; right graph); (**C**) Tibial subchondral bone plate

was selected as area of interest to perform analysis for *Lef1$^{fl/fl}$* and *ATC Lef1$^{fl/fl}$*. Upper two graphs show bone volume over tissue volume, BV/TV (%) for *Lef1$^{fl/fl}$* and *ATC Lef1$^{fl/fl}$* PTOA (left panel), and Sham (right panel). **(D)** Graphs represent subchondral bone plate thickness (µm^3), for Lef1fl/fl and *ATC Lef1$^{fl/fl}$* PTOA (left panel) and Sham (right panel). **(E)** Representative 2D µCT joint reconstructions of PTOA mice (n≥8 each, female n=4; male n=4; medial and lateral) and Sham medial (n≥8 each, female n=4; male n=4; and Sham lateral (n≥7 each, female n=3; male n=4). **(F**) 2D µCT joint reconstructions of sham *Lef1$^{fl/fl}$* and *ATC Lef1$^{fl/fl}$* mice were obtained and quantified for meniscal mineral content (µm^3) of the medial and lateral joint compartments. (**G**) Sham *Lef1$^{fl/fl}$* and *ATC Lef1$^{fl/fl}$* mice (n=8; female n=4; male n=4) were analyzed for Collagen type I immune-staining, and quantified for internal and external fields of the lateral menisci ("LM"; as shown in Fig. 5D, right scheme). All experiments were subjected to Kruskal-Wallis and post hoc Dunn's test to assess differences between multiple experimental groups, assuming a confidence level of 95% ($p < 0.05$). To examine statistical significance between mice genotypes PTOA, we employed Mann-Whitney U test, considering $p< 0.05$ (denoted *) to be statistically significant.

SI_16: Figure legends for SI_1-SI_15.
